# Unveiling the Benefits of Artificial Intelligence in Individual, Organizational Management, and the Health/Sector System

**DOI:** 10.1002/hsr2.71536

**Published:** 2025-12-27

**Authors:** Teferi Gebru Gebremeskel, Frank Romeo

**Affiliations:** ^1^ College of Health Sciences and Medicine Aksum University Aksum Ethiopia; ^2^ SBARRO Organization Temple University of Philadelphia Napoli Italy

**Keywords:** artificial intelligence, benefit, deep learning, healthcare sector

## Abstract

**Background:**

Artificial intelligence (AI) is transforming healthcare by improving care, efficiency, and cost benefit, yet limited research compares its benefits across individual, organizational, and system levels to guide effective integration.

**Aims:**

This review examines how the adoption of AI can benefit healthcare by improving individual outcomes, enhancing organizational efficiency, and strengthening the healthcare system.

**Methods:**

Two reviewers screened studies published up to December 2023 from four databases—a three‐dimensional framework identified AI benefits for individuals, organizations, and the broader health sector.

**Results:**

A total of 92 articles met the inclusion criteria, showing AI provides meaningful benefits across all dimensions. The individual benefits dimension is precise but often context‐specific, showing substantial gains in early detection, diagnosis, and monitoring. AI reduces the workload by 19%–50%, improves reading times by 21%–54%, and enhances cancer detection by 20% and treatment timing. The organizational benefits dimension encompasses AI‐optimized workflows and cost reductions, enhancing efficiency in clinical settings and workflow management. The health sector system dimension shows the most substantial evidence, highlighting improved patient outcomes, workflow efficiency, data access, training, and overall system advancement. Despite these advantages, challenges remain regarding data integrity, patient safety, and privacy under strict healthcare regulations.

**Conclusion:**

AI offers benefits at all levels, with the most substantial evidence being at the system level, demonstrating clear improvements in patient care and workflow. Individual and organizational gains are promising, though more validation is needed. Future adoption should prioritize cost–benefit analysis and system‐wide AI integration while evaluating impacts at all levels.

## 1 Introduction

Artificial intelligence (AI) has emerged as a transformative force across industries, leveraging vast amounts of relevant data to enhance performance, streamline operations, and drive innovation. In recent years, AI has advanced rapidly, delivering significant benefits, particularly in healthcare, where its impact is both profound and multifaceted [1, 2].

In clinical settings, AI has revolutionized healthcare delivery by automating routine medical processes, thereby enabling professionals to focus on critical tasks such as patient care and resource management [3, 4]. AI‐powered systems are increasingly used for early detection and diagnostic applications, offering faster and more accurate identification of conditions such as cancer, cardiovascular diseases, and neurological disorders [4, 5, 6]. These systems utilize machine learning algorithms to analyze medical images, patient histories, and genetic data, often outperforming traditional diagnostic methods in terms of speed, precision, and consistency. A prominent example is the use of AI in radiology, where advanced algorithms interpret imaging data from X‐rays, CT scans, and MRIs to detect abnormalities such as tumors, fractures, and internal bleeding [7]. These tools not only enhance diagnostic accuracy but also streamline radiology workflows by prioritizing urgent cases, generating preliminary reports, and reducing the time required for image analysis—ultimately improving patient outcomes and operational efficiency [8].

Beyond diagnosis, AI plays a significant role in organizational management within healthcare institutions. Predictive analytics help hospitals anticipate patient admission rates, optimize staffing, and manage supply chains more efficiently [9]. AI‐driven decision support systems help administrators with resource allocation, financial planning, and policy development, ultimately enhancing operational efficiency and patient outcomes [10].

Outside the clinical realm, AI continues to reshape the experiences of individuals and the strategies of organizations across various sectors. For individuals, AI enhances daily life through personalized digital assistants, mental health applications, and adaptive learning platforms [11]. These tools provide tailored recommendations, real‐time support, and customized educational experiences, enabling users to make informed decisions and enhance their well‐being.

Organizations across finance, manufacturing, retail, and creative industries are leveraging AI to gain a competitive edge. In finance, AI algorithms detect fraudulent transactions, automate compliance checks, and optimize investment portfolios [12]. Manufacturing firms utilize AI for predictive maintenance, process automation, and quality assurance, thereby reducing operational costs and minimizing downtime [13]. Retailers employ AI to analyze consumer behavior, personalize marketing campaigns, and manage inventory with greater accuracy. Creative industries benefit from AI‐generated content, design assistance, and audience analytics, enabling more engaging and efficient production workflows.

Economically, the integration of AI drives substantial value creation. By automating repetitive tasks and enabling data‐driven decision‐making, AI reduces labor costs, increases productivity, and fosters innovation. Businesses can scale their operations more effectively, respond to market changes with agility, and deliver enhanced customer experiences [14]. At a macroeconomic level, AI contributes to GDP growth, job transformation, and the emergence of new industries, although it also necessitates proactive strategies to address workforce displacement and ethical concerns [14]. AI's ability to solve complex problems and improve predictive accuracy is transforming decision‐making and improving lives. For instance, a study in Australia demonstrated that AI significantly reduces the cost of detecting lung cancer, from approximately $150,000 to just $300 per case [15]. Furthermore, Bohr and Memarzadeh suggest that implementing AI in healthcare could potentially result in a cost reduction of USD 150 billion by 2026 in the United States [16]. These dramatic cost savings not only highlight AI's potential to make healthcare more accessible and efficient but also underscore its broader economic impact by lowering the financial burden on healthcare systems and enabling earlier interventions that reduce long‐term treatment costs.

At the clinical level, hospitals are increasingly adopting AI technologies to improve diagnostic accuracy while reducing costs, intervention times, hospital stays, and radiologists' workloads. For example, a German study demonstrated that AI reduced reporting times for critical chest radiograph findings from an average of 80 min to just 35–50 min [17], while in the United States, a commercial algorithm used to prioritize intracranial hemorrhage cases shortened waiting times from 16 to 12 min per positive case [18]. These improvements directly benefit individuals by enabling faster diagnoses and more timely treatment. At the organizational level, AI is being integrated into Health Management Information Systems, as highlighted in a systematic review by Abebe [19], which explores applications such as predictive analytics for patient care, natural language processing for efficient documentation, and machine learning to support clinical decision‐making. These tools enhance operational efficiency, optimize resource allocation, and support strategic planning. However, the review also highlights challenges such as data privacy, system interoperability, and the need for training healthcare professionals—factors that must be addressed to realize AI's potential fully. Overall, these studies demonstrate how AI is transforming healthcare delivery, enhancing individual outcomes, and enhancing organizational capabilities. Although numerous studies have examined AI applications within specific domains such as clinical diagnostics, healthcare operations, and business automation, there is a clear gap in comprehensive reviews that integrate AI's benefits across individual experiences, organizational management, and the broader health sector. Existing research often focuses on isolated technical implementations, overlooking the interconnected impact of AI on personal well‐being, institutional efficiency, and systemic improvements in healthcare. To date, no systematic review has holistically evaluated AI's contributions across these three dimensions. This study addresses that gap by synthesizing current literature and case examples to provide a unified understanding of how AI enhances clinical accuracy, streamlines organizational workflows, and empowers individuals through personalized and accessible services, offering valuable insights for future research, policy development, and the responsible integration of AI.

## 2 Methods

### 2.1 Study Design

A systematic review assessed the benefits and advantages of AI in individuals, organizations, and healthcare facilities, focusing on clinical diagnostic accuracy, cost‐effectiveness, time savings, and reduced workload.

### 2.2 Search Strategies

**Figure 1:**
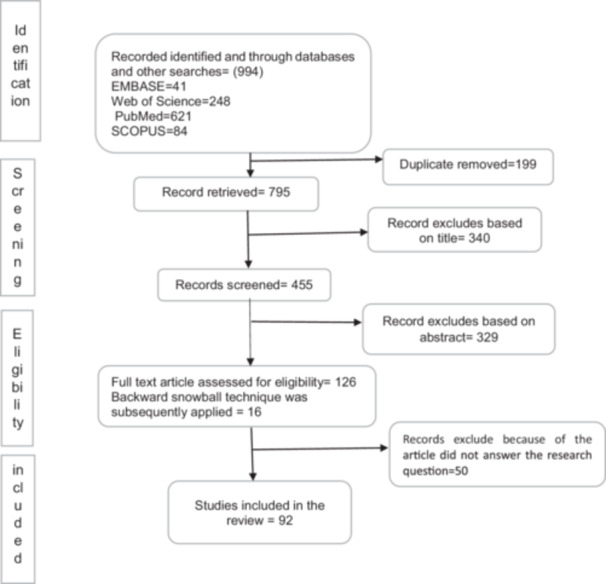
PRISMA flow diagram for screening process and outcomes.

To identify relevant literature published up to December 2023, we conducted a comprehensive search across multiple electronic databases. Initial keyword searches across PubMed, Web of Science, Scopus, and Emerald databases identified 994 unique articles. After applying database‐specific filters (such as publication type, language, and year), we conducted a manual screening process based on predefined inclusion and exclusion criteria. Manual filtering focused on topic relevance, study design, and methodological rigor. Specifically, we included empirical and conceptual studies that directly examined the role, applications, or benefits of AI in individual, organizational, or healthcare sectors. We excluded articles that were purely theoretical, non‐peer‐reviewed, or outside the scope of the study. Following this process, we reduced the total number of eligible studies to 455. Subsequent full‐text screening based on specific criteria further narrowed the selection to 126 articles. The backward snowball technique added 16 more, bringing the total to 142. Ultimately, a quality assessment led to the selection of 92 articles for analysis (Figure 1 details the database‐specific article distribution, with PubMed being the most significant source). To identify search terms, researchers begin by selecting unique words from recognized articles in the study area and utilize the advanced search features of selected databases to combine relevant search terms until they find all significant articles. The research study identified the following keywords: “artificial intelligence” OR “machine learning” OR “data processing” AND “healthcare” OR “medical center” AND “benefit” OR “advantage” OR “feature.” Data extraction was conducted in each database using the attached keyword (Supporting Information S2: Supplementary Material 1). Google Scholar and reference back searching were also used to identify additional literature.

### 2.3 Inclusion and Exclusion Criteria

We conducted this review in accordance with the Preferred Reporting Items for Systematic Reviews and Meta‐Analyses (PRISMA) guidelines to ensure transparency, reproducibility, and methodological rigor [20].

The review aimed to include all English‐language articles published up to December 2023, as well as scholarly articles and peer reviews that discussed the benefits or advantages of AI in healthcare or medical centers. Since we included only English‐language articles, there is a potential language bias, as relevant studies published in other languages may have been excluded.

Studies that provided one or more measures of the advantage or benefit of AI in healthcare or medical centers, such as likelihood ratio, cost‐effectiveness, saving time, and workload in their abstract, were incorporated in the review. We excluded abstracts from conferences and articles that did not report the outcome of interest.

We observed publication bias, as researchers overrepresented studies showing positive outcomes, underreported negative or inconclusive findings, and excluded gray literature or unpublished studies.

### 2.4 Data Extraction

We imported all retrieved studies into EndNote to exclude duplicates and titles that fulfilled the inclusion criteria. We performed data extraction using a standardized form developed specifically for this review. Before complete data extraction, the form was piloted on a sample of five studies to ensure clarity, consistency, and completeness of the data field. Based on this pilot, we made minor adjustments to improve usability and alignment among reviewers. After calibration, two reviewers independently extracted data from all included studies, resolving any discrepancies through discussion and collaboration. The initial identification and abstract screening were also conducted by two reviewers (T. G. G. and F. R.), ensuring that each article met the established methodological criteria. These same reviewers proceeded with the full‐text review, and we addressed any disagreements encountered during the screening process through discussion and a second round of full‐text evaluation.

This systematic review evaluated the validation methodologies and generalizability of AI in revolutionizing healthcare. Rather than independently validating the models, our analysis critically assessed the validation strategies employed within the primary studies included in the review. This study involved a systematic search for studies detailing internal validation techniques (e.g., cross‐validation, bootstrapping), noting the specific performance metrics used. The lack of external validation or limited data diversity significantly impacted our assessment of the generalizability of each study.

Our review synthesized these findings to provide a comprehensive overview of validation approaches and the generalizability of results across diverse settings. We explicitly addressed limitations within the primary studies, highlighting the absence of external validation or insufficient data diversity. This rigorous approach allowed for a nuanced and critical appraisal of the evidence, providing a clearer understanding of the robustness and generalizability of the AI models under examination. We extracted quantitative and qualitative data from all eligible articles using Microsoft Excel.

The data extraction form contains the first author's name, year of publication, country, setting, attributes of a given population intervention (index test), comparator(s), reference, outcome measurement, sample, design, analysis, and results for the outcome of interest. We evaluated each study that met the inclusion criteria using the STARD 2015 checklist to assess validity and potential bias [21].

A three‐dimensional approach established a systematic framework for organizing and interpreting data. This approach encompasses the advantages that organizations can derive from leveraging AI technology. First, there are benefits for individuals, including automated decision‐making, patient monitoring (with a focus on the elderly), early diagnosis, and streamlined processes [22, 23]. Second, there are benefits for organizations themselves, encompassing workflow assistance, performance enhancement, cost reduction, and fraud detection [3, 24]. Lastly, there are benefits for the broader sector, including time savings, reduced resource consumption, professional training, data sharing, and improved data accessibility [4, 25]. This study did not require ethical approval since it utilized published data. We found evidence of publication bias, as studies with disproportionately favorable outcomes were overrepresented, while those with negative or inconclusive results were underrepresented. Such selective reporting may overestimate the effectiveness of AI applications. Heterogeneity among studies—stemming from differences in AI models, applications, data sets, and outcome metrics—precluded formal meta‐analysis. Instead, we employed a narrative synthesis approach, enabling a descriptive comparison of results across domains. This approach allowed us to identify trends, strengths, and limitations without overgeneralizing findings across heterogeneous methodologies.

## 3 Results

Figure 1 illustrates the final number of articles selected for the present review study. Distribution of articles according to the classification framework: the research topic organizes itself into three dimensions: individual, organizational, and sector benefits.

Supporting Information S1: Table 1 displays the number of articles published each year in each dimension. Thus, the aggregate number of articles published under individual benefits is (*n* = 43), organizational benefits (*n* = 31), and sector benefits (*n* = 18). Most articles investigate the impact of AI on the healthcare sector, examining benefits at the individual, managerial, and sectoral levels (Supporting Information S1: Table 1).

Over the past decade, studies examining the benefits of AI to the industry have been relatively few. In contrast, the use of AI at the individual level has attracted moderate research interest and ranks as the third most studied topic in this classification framework. Meanwhile, the benefits of AI in enhancing organizational performance have received the most significant attention from researchers during this period.

### 3.1 Individual Benefit or Advantage

Studies showed that automated decision‐making [1, 3, 4, 5, 26, 27, 28, 29, 30, 31, 32, 33, 34, 35, 36, 37, 38, 39, 40, 41], patient and elderly monitoring [30, 40, 42, 43, 44, 45, 46, 47, 48, 49, 50, 51, 52, 53, 54, 55, 56, 57, 58, 59], and early diagnosis [22, 27, 28, 36, 41, 45, 54, 60, 61, 62, 63, 64, 65, 66, 67, 68, 69, 70, 71, 72], and process simplification [23, 36, 37, 39, 72, 73, 74]. AI notification can facilitate the early identification of incidental findings, not only in urgent cases.

For instance, AI can detect lung nodules and vertebral fractures on chest radiographs using automated algorithms. One CT‐based predictor analyzed scans from 48,967 patients in Israel, collected between 2010 and 2017. It accurately estimated each patient's 10‐year risk of fracture. The tool also identified individuals who may have been undertreated or underdiagnosed for osteoporosis [72]. These findings highlight the potential of AI to enhance early detection and risk stratification in clinical practice. For future adoption, this suggests that integrating such predictive tools could support more personalized patient care, optimize treatment decisions, and help address gaps in current diagnostic workflows.

AI enhances clinical alarm systems by accurately classifying patient deterioration [75], monitoring health decline in hematologic malignancies [76], identifying medication‐related issues, and predicting adverse events [77]. For example, in one study involving 2153 patients in a 24‐bed trauma step‐down unit, AI was used to monitor health decline in individuals with hematologic malignancies. A neural network was able to distinguish meaningful alerts from artifacts in real‐time vital sign data [76]. These findings suggest that AI can significantly improve the reliability and efficiency of clinical monitoring systems. For future adoption, this indicates the potential to reduce alarm fatigue, prioritize critical interventions, and support more proactive, patient‐centered care in high‐acuity hospital settings.

### 3.2 Organizational Benefit or Advantage

Organizations use AI applications and IT tools to reduce costs [24, 25, 37, 46], to detect fraud [16, 78, 79, 80, 81, 82, 83, 84], to improve performance [1, 3, 4, 29, 32, 40, 46, 61, 85, 86], and provide workflow assistance [3, 51, 87].

AI has the potential to enhance workforce efficiency significantly. A survey conducted by the Royal College of Radiology received responses from all radiology departments in the United Kingdom. Between 2013 and 2018, the number of CT imaging exams increased by 54%, and the number of MR exams rose by 48%. However, the radiology workforce grew by only 19% during the same period [86]. This gap underscores the need for AI to help meet the rising demand and enhance efficiency. In another survey of 675 members of the European Society of Radiology (ESR), representing 2.8% of the 24,000 contacted, 67% of respondents were male, and 82% worked in academic or public hospitals—notably, 50% of participants expected AI to reduce their reporting workload [88].

These findings suggest that AI adoption could play a crucial role in addressing workforce shortages and managing the increasing demands for imaging. For clinical practice, this underscores the potential for AI to streamline radiology workflows, reduce clinician burnout, and support timely patient care, particularly in high‐volume settings.

### 3.3 Sector Benefit or Advantage

Studies revealed that time‐saving [3, 22, 24, 37, 38, 39, 89, 90], reduction of resource consumption [5, 25, 91], professional training provision [29, 92, 93, 94, 95, 96, 97, 98, 99], industry‐wide data sharing [29, 100, 101, 102], and industry‐wide data availability [67, 103, 104, 105].

In a large clinical trial [106] involving 80,033 women, participants were randomly assigned to 2 groups. The intervention group, with 40,003 women, received AI‐supported breast cancer screening. The control group, consisting of 40,030 women, underwent standard double reading without AI assistance. The AI‐supported group required 44% less radiologist workload. It also detected 20% more cancers compared to the standard screening group. These results highlight the transformative potential of AI in clinical practice, suggesting that AI‐assisted screening can both improve diagnostic accuracy and significantly reduce clinician burden. For future adoption, this implies that integrating AI tools could optimize resource allocation in high‐volume screening programs, accelerate early cancer detection, and ultimately enhance patient outcomes.

Martini et al.'s [89] study, conducted among 100 patients (median age, 60 years; range, 19–80 years), found that using vessel suppression techniques in CT thorax imaging resulted in a significant 21% decrease in the time required to identify pulmonary metastases.

These findings suggest that AI‐assisted image processing can streamline diagnostic workflows and reduce clinician workload, potentially allowing faster treatment decisions. For future AI adoption, this indicates that integrating such techniques could enhance efficiency in radiology departments, particularly for high‐volume or time‐sensitive cases, while also improving patient outcomes by enabling earlier detection and intervention.

In a German hospital study [17], researchers used 10,000 chest X‐rays to train and test an AI system—8000 for training and 2000 for testing. The study examined how AI affected report turnaround times (RTAT) for critical findings using different prioritization methods. The findings revealed that AI‐driven prioritization significantly improved response times for urgent cases, highlighting AI's potential to optimize radiology workflows and enhance patient safety. By accelerating the detection of life‐threatening conditions, such as pneumothorax, AI could serve as a critical triage tool—particularly in high‐volume settings—supporting radiologists in making faster, more accurate decisions and reducing diagnostic delays in emergency care.

Compared to the traditional first‐in, first‐out (FIFO) approach, AI‐based prioritization significantly reduced average RTAT for critical cases. For example, pneumothorax cases showed a faster average RTAT: 35.6 min with AI versus 80.1 min using FIFO.

While the maximum RTAT increased in some cases (1293 min vs. 890 min), applying an “upper limit” approach brought this down to 979 min—still better than FIFO's 1293 min. These findings suggest that AI can meaningfully enhance patient safety and clinical efficiency by ensuring that critical cases are addressed more quickly. For future AI adoption, this indicates a potential shift in workflow prioritization strategies, with an emphasis on real‐time decision support over traditional queue‐based approaches. Clinically, it underscores the value of integrating AI tools to optimize resource allocation, while also highlighting the need for safeguards to prevent extreme delays in less urgent cases.

Applying a commercial algorithm to 4510 head CT exams from 3788 US patients improved outcomes. The AI prioritized cases of intracranial hemorrhage, reducing waiting times from 16 min to 12 min per positive case [18].

This demonstrates AI's potential to speed up medical image interpretation and reporting. Beyond efficiency, such advancements suggest a shift toward faster triage and earlier interventions in emergency care—potentially reducing complications, improving survival rates, and easing clinician workload.

These results reinforce AI's growing role as a decision‐support tool, indicating a potential for broader integration in radiology workflows and emergency medicine in the near future.

According to Svoboda [90], using AI on 42,290 chest CT scans from 15,000 patients helped detect tumors earlier. This could give oncologists twice the treatment time, improving the chances of preventing cancer spread.

Faster diagnosis enables quicker treatment planning, ultimately improving patient outcomes and quality of life. These findings underscore AI's potential not just as a diagnostic aid, but as a transformative tool in oncology—shifting care from reactive to proactive. By enabling earlier intervention, AI may redefine clinical workflows, support precision medicine, and strengthen efforts to personalize care while reducing the burden on healthcare systems.

### 3.4 Comparative Summary of AI Benefits Across Dimensions

Table 1 summarizes the three dimensions with evidence strength, trends, and gaps. AI shows meaningful advantages across individual, organizational, and sector levels. At the personal level, it supports early detection, risk stratification, and patient monitoring, such as identifying lung nodules, vertebral fractures, and patient deterioration, enabling personalized care and proactive interventions; however, most evidence comes from single‐center studies. At the organizational level, AI can enhance workflow efficiency and reduce radiologist workload, addressing the gap between rising imaging demand and workforce growth; however, much of the evidence is survey‐based rather than outcome‐driven. The sector or health system level has the most substantial proof, with large‐scale studies demonstrating faster triage, improved RTAT, earlier cancer detection, and overall workflow optimization. At the same time, gaps remain in cost‐effectiveness analyses, integration across diverse settings, and safeguards for less urgent cases.

**Table 1:** Summarizing the three dimensions with evidence strength, trends, and gaps.

Dimension	Key evidence/findings	Trends	Gaps/limitations
Individual	– AI detects nodules, vertebral fractures, and patient deterioration. – Supports early risk stratification and proactive interventions.	– Personalized care and patient monitoring are improving. – Reduced alarm fatigue and faster clinical decision‐making.	– Mostly single‐center or condition‐specific studies. – Need for broader, multicenter validation.
Organizational	– AI can reduce workload by ~50%. – Addresses gaps between rising imaging demand and workforce growth.	– Workflow efficiency and clinician support are increasing. – AI adoption expected to reduce burnout and improve throughput.	– Evidence largely based on surveys rather than measured outcomes. – Limited objective, large‐scale organizational impact studies.
Sector/health system	– Large trials show faster triage, improved RTAT, earlier cancer detection. – AI enhances patient safety, clinical efficiency, and population‐level outcomes.	– System‐level integration is growing. – Supports early interventions and optimized resource allocation.	– Need for cost‐effectiveness studies. – Challenges in integrating AI across diverse healthcare settings. – Safeguards required to prevent delays for less urgent cases.

## 4 Discussion

This systematic review included 92 articles that met the predefined inclusion criteria. The review adopted the Swim (Synthesis Without Meta‐analysis) reporting guideline, which provides an established framework for synthesizing findings when it is not feasible to pool quantitative data. The studies covered diverse healthcare settings across multiple countries, each influenced by varying clinical practices, unit costs, healthcare delivery models, and analytical perspectives. A notable gap emerged regarding the economic and organizational feasibility of implementing these technologies, particularly in terms of potential increases in healthcare spending and the need for additional resources such as staff training and change management—topics that were largely absent from the reviewed studies. Such insights are crucial for decision‐makers to assess integration scenarios and tailor strategies to their specific institutional contexts.

We also noted methodological limitations. Many studies have used a 1‐year time horizon without justification, which may overlook long‐term costs, especially in populations with chronic diseases. Others relied on single RCTs, limiting generalizability to routine clinical practice. However, these details are crucial for informing cost analysis and ensuring relevance across various healthcare settings. Improving transparency in cost reporting would provide stakeholders with a clearer understanding of financial implications.

AI has enabled early diagnosis in oncology, cardiology, and the detection of infectious diseases. However, Kitsios et al. [106] cite that such applications are sensitive to changes in input distributions—for example, due to new imaging devices or updated clinical protocols.

### 4.1 Advantage for Individuals

Our review underscores AI's transformative role in advancing individualized care by effectively analyzing large, complex medical data sets to improve diagnosis, treatment planning, and patient outcomes. However, most supporting evidence comes from controlled or single‐institution studies, which limit its generalizability across diverse healthcare contexts. While AI demonstrates strong technical performance, real‐world implementation often reveals vulnerabilities—particularly model degradation over time as healthcare systems, populations, and technologies evolve. Models trained on narrow demographic or geographic data sets, for instance, may perform poorly in different patient groups, reflecting an underlying lack of data diversity and external validation [107]. Transitions between electronic health record (EHR) systems further expose limitations, as inconsistencies in coding, documentation, or data formats can compromise accuracy and trigger false alerts [108]. Moreover, rapid medical innovation—such as the introduction of new devices and diagnostic methods—can quickly render existing models obsolete [37]. These gaps highlight the need for longitudinal, multicenter validation and adaptive retraining protocols to ensure sustained accuracy, mitigate bias, and preserve clinician trust. Without systematic updating and governance, AI risks amplifying inequities and undermining the very goals of precision and safety it seeks to achieve.

### 4.2 Advantage for the Organization

AI models in healthcare demonstrate strong potential for enhancing prediction and decision‐making, yet their reliability depends on how effectively they adapt to evolving populations and technologies. Evidence supports their effectiveness in controlled settings; however, most studies rely on retrospective or single‐institution data, which limits their external validity. As demographics, disease prevalence, and care practices evolve, models trained on outdated or homogeneous data sets risk embedding bias and misclassifying new patient groups. The COVID‐19 pandemic further exposed these limitations, as many pre‐pandemic models failed to account for novel clinical features or rapidly shifting healthcare demands.

Technological transitions—such as the adoption of new EHR systems—add further complexity to the process. Variations in data structure, coding, and interoperability can degrade model accuracy and restrict generalizability. Although tools like the Epic Deterioration Index highlight AI's potential for early detection, their performance is often context‐dependent and rarely validated across diverse environments. Future work must prioritize adaptive frameworks, continuous retraining, and cross‐institutional validation to sustain accuracy, minimize bias, and ensure equitable clinical application.

### 4.3 Advantage for Sectors

AI is transforming healthcare by improving diagnostics, treatment planning, and operational efficiency. Substantial evidence demonstrates its ability to enhance patient care, accelerate research, and streamline workflows. However, much of this evidence comes from small‐scale or single‐center studies, which limit its generalizability across diverse healthcare settings. AI performance also tends to decline over time as populations, technologies, and clinical practices evolve—posing challenges for long‐term reliability. Models trained on static or homogeneous data sets often fail to capture emerging disease trends, shifting demographics, or new diagnostic standards, leading to potential bias and reduced accuracy. While adaptive strategies such as federated learning, continuous retraining, and human‐in‐the‐loop validation can mitigate model drift, their large‐scale implementation remains limited by data‐sharing barriers, cost, and governance issues. Frameworks like the Personal Health Train (PHT) [99] offer promise for secure and interoperable data exchange; however, real‐world validation remains scarce. Future research should focus on evaluating these adaptive systems in multi‐institutional, real‐time contexts to establish robust, equitable, and sustainable integration of AI in healthcare.

### 4.4 Challenges

Several challenges may deter organizations from using AI. Machine learning, natural language processing, and expert systems utilize medical data as input to process and create models that support medical decisions within healthcare systems. Most applications of AI in healthcare systems are related to diagnosis and treatment. False decisions in automated diagnosis can have extremely harmful consequences. Collected data from hospitals are sometimes not of sufficient quality or simply inaccurate. Data errors are among the top challenges in medical data processing using AI [109, 110, 111, 112]. Another challenge is the decision errors made by machine learning algorithms. Sometimes, the applied algorithm is unsuitable for the given data, or the data are not sufficiently reliable for classification algorithms such as neural networks, decision trees, and Bayesian networks. Several studies have demonstrated possible decision‐making problems in the health domain and their solutions [32, 113, 114]. However, not all of them are automated: doctors make the final decision, and this interplay between healthcare practitioners and AI models may result in false diagnoses and treatment results [37, 53, 115].

Some AI methods require a large volume of data to process. Collecting data, especially patient data, can be challenging due to the ethical implications associated with such data. Applying some classification and clustering algorithms to a minimal amount of data may yield perfect accuracy; however, this may not be realistic or applicable [29, 116, 117]. We need to preprocess the collected data to use it in AI techniques. Text data require significant natural language processing before it can be used effectively. Different data types, such as text, numeric, image, and video, must sometimes be integrated using the same algorithm, which is one of the most challenging tasks in medical data processing [29, 116, 117]. Researchers and healthcare professionals can collect medical data from various sources and formats, including medical images, 3D video sequences, photographs, and numeric data. However, they face challenges in gathering clean, robust, and efficient data for healthcare data analysis.

There are obvious privacy issues related to accessing, editing, sharing, and using patient data. Cloud computing and AI play a crucial role in various healthcare applications. These systems collect, process, store, monitor, and share health data [22, 118, 119]. Despite the advantages of these systems, several challenges also exist, including security issues, privacy implications, cybersecurity concerns, and ethical considerations. Hospitals and government agencies typically have established ethical procedures for collecting and sharing data.

Permission is required from a government‐approved authority to collect and use data, even for research purposes [120]. Other ethical issues associated with AI in healthcare and broader sectors include inequality, unemployment, human rights, commitment to a cause, regulatory approaches, behavioral biases, population biases, and linking biases [119]. To mitigate ethical issues in AI applications in the healthcare sector, studies have focused on minimizing adverse side effects, reward hacking, safe exploration, and robustness [119, 121, 122]. Many doctors use machine learning algorithms for the early prediction, treatment, and diagnosis of diseases, as these algorithms can make or support their decision‐making. Governments have expressed concerns about whether these automated processes adequately protect patients' rights. Such concerns have led to several regulations in data collection, processing, technology usage, and quality, as well as collection and analysis methodologies. In addition, researchers working in the healthcare domain should carefully focus on data quality, data testing, and documentation [100, 123] before AI applications can utilize them.

### 4.5 Limitations of This Review

Our review has the following limitations. (i) It was challenging to access specific details about the functioning of AI operations due to the proprietary nature of these functionalities, which often need to be fully disclosed in academic papers. (ii) We implemented a thorough search strategy; the review should have included certain studies on AI in healthcare, such as those in gray literature and reports not part of the selected databases.

### 4.6 Implication and Future Directions

Our review offers a comprehensive understanding of the benefits of AI in healthcare, encompassing individual, organizational, and sectoral levels. It highlights how AI can enhance patient care and treatment by analyzing complex medical data effectively, leading to innovative solutions. Integrating AI with health coaching shows promise in managing chronic diseases and reducing healthcare costs. Organizations can benefit from AI applications in several ways, including cost reduction, fraud detection, performance improvement, and workflow assistance. AI also offers opportunities for resource optimization and cost reduction in the healthcare sector. Implementing privacy‐focused infrastructure, such as the PHT system, can address privacy and data access challenges. Integrating AI into medical education can enhance educational endeavors and equip future healthcare professionals with the knowledge and skills necessary for AI‐related applications. Ultimately, AI has the potential to bring holistic benefits to the entire healthcare sector, driving further research and development efforts to improve healthcare outcomes and patient‐centered care.

Future directions involve advancing AI technologies, implementing AI in chronic disease management, formulating effective AI adoption strategies, optimizing resources, addressing privacy challenges, integrating AI into medical education, and driving comprehensive implementation of AI in healthcare through interdisciplinary collaborations. These efforts aim to enhance healthcare outcomes and promote patient‐centered care while fostering innovation.

## 5 Conclusion

While AI demonstrates meaningful advantages at all levels, the sector/health system dimension shows the most robust and large‐scale evidence, with tangible patient and workflow outcomes. Individual benefits are clear but often context‐specific, and organizational benefits are promising but require more objective evaluation. Some challenges include data integration, privacy, legal, and patient safety. This review study presents implications for practice, guiding future studies and decision‐makers in the healthcare sector. It is crucial to assess whether the benefits of AI outweigh the challenges associated with its application in the healthcare sector. According to the literature analyzed in this review, there is sufficient evidence to believe that AI can significantly benefit the healthcare sector. However, the challenges related to real and perceived data integrity, as well as the resultant patient safety and privacy issues associated with using AI in healthcare, must be carefully evaluated, primarily due to the strict regulations governing the healthcare sector. Many organizations are updating policies to protect patient data and ensure confidentiality. Further research is needed to investigate the factors influencing AI adoption in healthcare. The applications and benefits discussed here can be explored in future studies using varied methods. Adoption strategies should focus on system‐level integration while assessing impacts at both individual and organizational levels.

## Author Contributions

Teferi Gebru Gebremeskel designed and drafted the review. Teferi Gebru Gebremeskel and Frank Romeo were screened based on eligibility criteria, and the final paper was read and approved.

## Disclosure

The lead author Teferi Gebru Gebremeskel affirms that this manuscript is an honest, accurate, and transparent account of the study being reported; that no important aspects of the study have been omitted; and that any discrepancies from the study as planned (and, if relevant, registered) have been explained.

## Ethics Statement

Since this study utilized published data, no ethical approval is required.

## Consent

Informed consent statement is not applicable.

## Conflicts of Interest

The authors declare no conflicts of interest.

## Supporting information

Table 1: integration of AI impacts the reading times of radiologists during the daily interpretation of chest X‐ray studies of the included papers.

Supplementary Material 1.

Supporting Information S1 PRISMA.

## Data Availability

The authors have nothing to report.
