# Supplementary material for: Unveiling the Benefits of Artificial Intelligence in Individual, Organizational Management, and the Health/Sector System
Source: Health Sci Rep. 2025 Dec 27;9(1):e71536. doi: 10.1002/hsr2.71536 (PMC12743724; doi:10.1002/hsr2.71536)
Supplement: Supplementary file 1 — Table 1: integration of AI impacts the reading times of radiologists during the daily interpretation of chest X‐ray studies of the included papers. [file HSR2-9-e71536-s003.docx]

Table 1: integration of AI impacts the reading times of radiologists during the daily interpretation of chest X-ray studies of the included papers.

| Author, country, and year. | Study aim | Attributes of a given population | AI algorithm intervention (Index test) | Outcomes and result | Remark |
| --- | --- | --- | --- | --- | --- |
| Bernardini, M., et al 2021 (1). Italy | To propose a novel Semi-Supervised Multi-Task Learning (SS-MTL) approach for predicting short-term KD evolution on multiple General Practitioners’ EHR data. | **Input**: Labelled and Unlabelled training sets.  Pathology: Kidney disease.  **Semi-Supervised Learning**: to utilize both labelled and unlabelled patient data.  **Multi-Task Learning**: utilize to predict multiple outcomes  **Measures:** Accuracy, Macro-precision, Macro-recall, Macro-F1 and AUC. | Machine learning | The SS-MTL approach was mostly capable to gain useful information from unlabelled patients when labelled patients are less numerous than those unlabelled. | The real-case general practice scenario, where available labelled samples are limited, but those unlabelled are much more abundant. The SS-MTL approach, exhibiting also a high level of interpretability (i.e., age and pharmacological pathway were the most important predictors), might be the ideal |
| Yu, H. and Z. Zhou, 2021(2). China | To optimize IoT-based artificial intelligence-assisted telemedicine health analysis systems. | **Input:** Multimodal physiological and environmental data.  **Study design**: Experimental evaluation of a cloud-converged health IoT  **Sample size:** N/A  **Quality of Service Assurance:** computer simulations. | AI | Computer simulation, showing improved data reliability, sensing accuracy, and AI-driven emotional interaction. Its QoS framework enhances emotion recognition and physiological data collection. | The results highlight the potential of AI-assisted telemedicine but emphasize the need for real-world validation. |
| Dhieb, N., et al(3) 2020. | A Secure AI-Driven Architecture for Automated Insurance Systems: Fraud Detection and Risk Measurement. |  Population**:** focuses on **auto insurance claims.**  **Input:** Dataset **insurance claim records.**  **Sample Size:** N/A  ** Study Design:** Comparing fraud detection models and integrating **blockchain technology.**   Measurements**: accuracy, precision, recall, and F1-score.** | **Machine learning** | XG Boost detects fraud 7% more accurately than decision trees. AI models predict risk levels and future claims. Blockchain secures transactions and prevents fraud.  VFDT improves real-time fraud detection. | This **new framework** enhances efficiency and **reduces financial losses** in the insurance sector. |
| Sqalli, M.T. and D. Al-Thani, 2019(4). Quatar | introduce a model for health coaching empowered by the use of Persuasive Technology with a focus on AI | **Population:** Patients with chronic diseases.  **Input:** AI-driven health coaching interventions  **Sample size:** N/A  **Study design and period:** Literature review and model proposal;  **Measurements:** Effectiveness of AI-supported health coaching | **AI** | AI-driven coaching boosts long-term care adherence by offering personalized feedback and motivation. Unlike episodic visits, AI enables continuous, adaptive support for chronic conditions, promoting healthier habits while fostering patient autonomy and clinician collaboration. | underscores the importance of integrating AI into health coaching rather than relying solely on traditional healthcare visits.  It highlights the need for structured AI interventions that balance automation with human interaction. |
| Zhou, L. et al(5).,2020. | To develop a fully automatic, rapid, and accurate segmentation and quantification method for detecting COVID-19 infection regions in CT scans. | **Population:** COVID-19 patients  **Inputs:** CT scan images  **Sample size**: Utilizes a multi-country, multi-hospital, and multi-machine dataset  **Study design:** Experimental study using AI-based segmentation techniques  **Measurement**: Accuracy | Novel Deep Learning Algorithm | The proposed method significantly improves segmentation accuracy compared to existing approaches.  The CT scan simulator effectively mitigates data scarcity issues, enhancing model robustness.  The novel deep learning algorithm reduces complexity while maintaining high precision. | The study demonstrates strong applicability across different hospitals and imaging machines, making it a machine-agnostic solution for COVID-19 diagnosis. |
| Dagan, N., et al(6).,2020 Israel | To develop an automatic, opportunistic fracture risk evaluation method using routine abdomen or chest CT scans, addressing the underutilization of traditional methods like DXA and FRAX | **Population**: aged 50-90 years (51.8% women) with available CT scans before 2012  **Inputs:** Vertebral compression fractures (VCFs), Simulated DXA T-scores, Lumbar trabecular density, CT metadata (age and sex)  **Sample size**: 48,227 individuals  **Study design**: Retrospective cohort study  **Measurement:** (AUC), sensitivity, and positive predictive value (PPV), Fracture risk predictions using FRAXnb and the CT-based predictor | Machine learning | The CT-based predictor outperformed FRAXnb for major osteoporotic fractures: +1.9% AUC, +2.4% sensitivity , and +0.7% PPV.  For hip fractures, the CT-based predictor was noninferior to FRAXnb within a 1% margin. | When FRAXnb inputs are unavailable, fracture risk can be automatically evaluated using a single abdomen or chest CT, which is often readily available for screening |
| Sasu Billi, S.M.2020(7). India and USA | To implement machine learning techniques, particularly **random forest** and **Truth Maintenance System (TMS)**, in healthcare. | **Population:** Individuals with various health condition  **Input:** Medical records, symptoms, and historical health data  **Measurements:** Performance evaluation of random forest and TMS algorithm, assessing their accuracy and reliability | Machine learning | Random forest emerged as the most accurate model in predicting disease patterns and health risk factors.  The TMS algorithm enhances trust in the machine learning process  Machine learning can be strategically applied to healthcare, though not universally suitable for all medical conditions. | Random forest was the most accurate in identifying disease patterns, but it does not provide a detailed comparison with other models or justification for its superiority. |
| Kaur, A.,etal 2021(8). **India, Kenya, Ghana, Zambia, and Vietnam.** | To explore the challenges and opportunities of using AI and big data analytics in resource-poor healthcare systems | **Population: Healthcare systems in resource-poor countries.**  **Input: Medical imaging, patient records, pharmaceutical reports, and medical devices.**  **Study design: review-based analysis**  **Measurement:** evaluates healthcare performance | AI | AI has demonstrated superior performance in diagnosing diseases like cancer, heart conditions, and radiology-related issues.  Resource-poor countries face barriers  Ethical concerns, transparency, and accountability remain significant hurdles in AI adoption. | Big data analytics can help structure and analyse medical data to improve healthcare practices, but implementation requires careful ethical considerations |
| Thakkar, B.A.,2010(9). India | To develop an intelligent swine flu prediction system | **Population: Patients suspected of having swine flu**  **Input: 17 symptoms of swine flu recorded**  **Sample Size: 110 symptom sets**  **Study Design & Period: Prototype development and evaluation study;**  **Measurements: Used a Naïve Bayes classifier** | Machine Learning | -The model achieved an **accuracy of 63.33%** in predicting swine flu cases.  - Implemented on the **JAVA platform**, demonstrating feasibility for real-world applications. | -The potential of machine learning in medical diagnostics but acknowledges the need for further refinement to improve accuracy. |
| Moein, M., et(10) al.2010. Iran | To develop a medical decision-support system for diagnosing ear disorders. | **Population:** Patients with ear disorders.  **Input: Patient symptoms and diagnostic data related to ear disorders.**  **Study Design: classification-based analysis**  **Measurement: Classification accuracy using MLP neural networks and SVM.** | Machine Learning | -SVM outperformed MLP neural networks in classifying high-dimensional data related to ear disorders. | -The potential of machine learning in improving diagnostic accuracy and reducing medication errors.  -AI-driven classification models can enhance healthcare efficiency and patient safety. |
| O'neill, T.J. et al.2020(11). USA | To optimize the delivery of machine learning techniques in a clinical setting. | **Population:** Patients undergoing non-contrast-enhanced CT scans.  **Input:** CT scan images  **Study period:** September 2017 and March 2019.  **Measurements:** Queue-adjusted wait time and turnaround time. | AI | -Reprioritization of flagged cases led to a reduction in queue-adjusted wait time (from 15.45 minutes to 12.02 minutes).  The greatest improvement was observed in routine inpatient and outpatient cases, which typically have lower priority. | -The importance of how AI-generated flags are presented to radiologists in optimizing workflow efficiency. |
| Kim et al. (12)2020, South Korea | -To assess the AI's ability to identify pneumonia and visible pneumonia on CRs, comparing its performance with that of physicians. | No of the case: 387  Pathology: pneumonia  Test observer: 3 Physicians  Image modality: chest x-ray | Machine Learning | 165 min Without AI-Based CAD (time) Vs 101 min with AI-Based CAD (time) | The use of the DL algorithm in interpreting chest X-rays has shown remarkable benefits in terms of reducing reading time for ED physicians. |
| Liu et al(13). 2020 | How AI trilogy enhances medical care, reduces quarantine processing times through smart quarantine station diversion, AI-assisted image interpretation, and a clinical decision-making algorithm | Pathology: COVID-19  Pneumonia  No of the case: 643  Image modality: chest x-ray | Machine Learning | 153 min (station survey time) Without AI-Based CAD (time) Vs 35 min (station survey time) With AI-Based CAD (time) | The integration of an AI application in the quarantine station led to a substantial decrease in survey time, enhancing efficiency in patient evaluation without a clear indication of its impact on diagnostic accuracy. |
| Osareh, A. and B. Shadgar,2010(14)  Iran | To enhance breast cancer diagnosis using machine learning techniques | Population: Breast cancer patients  Inputs: Various machine learning classifiers  Study design: computational study leveraging machine learning models  Sample size: two widely used breast cancer benchmark datasets  Measurements: diagnostic accuracy | Machine Learning | By combining classifiers like Support Vector Machines (SVM), K-Nearest Neighbours (KNN), and Probabilistic Neural Networks (PNN) with feature selection methods, the researchers achieved high diagnostic accuracy of 98.80% and 96.33% using SVM models on two benchmark datasets. | The combination of feature selection and machine learning classifiers suggests that AI-driven approaches can play a crucial role in improving early detection and classification of breast tumours. |
| Zhang et al(15). 2021 | To showcase how AI at National Cheng Kung University Hospital enhances medical care and reduces quarantine processing times. | Pathology: COVID-19  Pneumonia  No of the case: 15  Image modality: CT  Test observer: 2 physicians.  (13–15 years  experience) | Deep Neural  Network algorithm | 3.623 min (total reading time for multiple cases) Without AI-Based CAD (time) Vs 0.744 min (total reading time for multiple cases) With AI-Based CAD (time) | The substantial decrease in average diagnosis time, highlights the capability of AI to accelerate the diagnostic process. The study underscores the promising impact of artificial intelligence in reshaping medical imaging protocols and advancing healthcare delivery. |
| Ladgham, A., et al.2013. (16)Tunisia | To improve brain tumour recognition in MR images using an optimized Support Vector Machine (SVM) model | Population: Patients with brain tumours  Inputs: SVM classifiers, optimized with SFLA-based feature selection  Study design: A computational study  Measurements: classification accuracy | Machine learning | **SFLA-SVM** approach enhances tumor detection accuracy by optimizing feature selection and kernel parameterization in SVM learning. | The potential of machine learning in medical imaging, offering an efficient and automated approach to brain tumor classification. |
| Kozuka et al(17). 2020 | Assessing a deep learning CAD system's efficacy in detecting pulmonary nodules on CT by comparing radiologists' readings with and without CAD. | Pathology: Pulmonary nodule.  No of the case: 120  Image modality: CT  Test observer: 2 radiologists!  (1–4 years’ experience) | Faster Region-  Convolutional  Neural Network | 373 min Without AI-Based CAD (time) Vs 331 min with AI-Based CAD (time) | that vessel suppression in CT imaging could lead to more efficient and time-saving interpretations, potentially enhancing diagnostic workflows in oncologic imaging practices. |
| Razaak, M.et al 2014(18). **UK & Italy** | To evaluate the performance of seven state-of-the-art video quality metrics. | Population: Medical experts  Inputs: Various video quality metrics  Study Design: A computational study  Measurements: Correlation between video quality metrics and medical experts' | Machine learnings | VIF, SSIM, and UQI showed strong correlation with expert evaluations, confirming their reliability in assessing diagnostic video quality. | The importance of video compression standards like HEVC in maintaining diagnostic integrity while reducing storage and transmission costs. |
| Liu et al(19). 2019 | Assessing a deep learning CAD system’s efficacy in detecting pulmonary nodules on CTby comparing radiologist’s reading with and without CAD. | Pathology: Pulmonary nodule.  No of the case: 271  Image modality: CT  Test observer: 2 radiologists.  (10 years’ experience) | Dense Net and Faster  Region-Convolutional  Neural Network | 15 min Without AI-Based CAD (time) Vs 5–10 min with AI-Based CAD (time) | that vessel suppression in CT imaging could lead to more efficient and time-saving interpretations, potentially enhancing diagnostic workflows in oncologic imaging practices. |
| Gayathri, B. and C. Sumathi, 2015. (20)  India | To detect breast cancer risk using fuzzy logic, aiming to reduce the number of variables for faster diagnosis. | Population: Breast cancer patients  Inputs: Feature selection using LDA and classification through the Mamdani Fuzzy Inference Model  Study Design: A computational study  Measurements: accuracy, | UCI Machine Learning | The Mamdani fuzzy model effectively detects breast cancer risk with high accuracy, demonstrating the potential of fuzzy logic in medical diagnostics. | This study highlights the importance of data mining and AI-driven approaches in improving disease detection and reducing diagnosis time. |
| Martini et al(21). , USA. | To evaluate the impact of vessel suppression (VS) on nodule detection rate, inter-reader agreement, and reading time in oncologic chest computed tomography (CT). | Pathology: Pulmonary consolidations/  Nodules  No of the case: 100  Image modality: MDCT  Test observer: 2 senior radiologists,  2 final-year residents,  and 2 inexperienced  residents | Machine Learning | 194 s Without AI-Based CAD (time) Vs 154 s AI-Based CAD (time) | that vessel suppression in CT imaging could lead to more efficient and time-saving interpretations, potentially enhancing diagnostic workflows in oncologic imaging practices. |
| Zhou, Y., et al., 2018(22). China | To classify malignant and benign breast tumours using shear-wave elastography (SWE) data. | Population: Breast cancer patients.  Sample Size: 540 breast tumour images (318 malignant, 222 benign).  **Inputs:** Shear-wave elastography (SWE) images.  Study Design: A segmentation-free radiomics approach | Machine learning | Accuracy (95.8%), Sensitivity (96.2%), and Specificity (95.7%) in tumour classification. | the potential of AI-driven radiomics in clinical computer-aided diagnosis, offering a segmentation-free and highly accurate method for breast cancer detection. |
| Nam et al(23). 2021 | To assess deep learning algorithm influence on diagnostic accuracy, reporting efficiency, and workflow effectiveness. | Pathology: Pneumonia,  pulmonary edema,  active tuberculosis,  interstitial lung  disease, nodule/mass,  pleural effusion, acute  aortic syndrome,  pneumoperitoneum,  rib fracture,  pneumothorax,  mediastinal mass.  No of the case: 202  Image modality: Chest-xray  Test observer: 2 thoracic radiologists,  2 board-certified  radiologists, and  2 residents | Deep learning-based  algorithm (Deep  convolutional  neural network) | 2771.2 s Without AI-Based CAD (time) Vs 1916 s AI-Based CAD (time) | The deep learning algorithm enhances radiologists' efficiency and reduces reporting time for critical and urgent cases. |
| Saxena, S. and S. Prasad.2019 (24).India | To explore the effectiveness of machine learning and deep learning methods in predicting and detecting cancer | Population: Cancer patients.  Study Design: A computational study.  Measurements: prediction accuracy | Machine Learning & Deep learning | The study evaluates prediction accuracy improvements (20-30%) in cancer progression, recurrence, and mortality.  Unsupervised learning techniques can enhance cancer detection by automatically extracting features from diverse cancer datasets. | This study highlights the potential of machine learning-driven cancer diagnostics, offering a more interpretable and scalable approach compared to traditional Artificial Neural Networks (ANNs). |
| Sung et al(25). 2021, South Korea | To assess observer performance in identifying and pinpointing significant abnormalities on chest radiographs with and without the aid of a deep learning-based detection (DLD) system in a randomized crossover setup. | Pathology: Nodules,  consolidation,  interstitial opacity,  pleural effusion,  pneumothorax of the case: 128  Image modality: Chest-X-ray  Test observer: 2 thoracic radiologists,  2 board-certified  radiologists,  1 radiology resident,  and 1 non-radiology  resident | Machine Learning | 24 s without AI-Based CAD (time) Vs 12 s AI-Based CAD (time) | The deep learning algorithm enhances radiologists' efficiency and reduces reporting time for critical and urgent cases. |
| Zhang, Y., et al. 2020, (26)  China | To improve deep learning-based medical image diagnosis | Population: Medical images  Inputs: UDA techniques  Study Design: A computational study analyzing.  Measurements: The study evaluates generalization performance and classification accuracy | Machine Learning | The Collaborative UDA algorithm improves domain adaptation by handling label noise and transferability, demonstrating superior performance. | This study highlights the potential of UDA in medical imaging, offering a robust and scalable approach for improving diagnostic accuracy. |
| Beyer et al(27). 2007, USA | Comparing AI with traditional visual diagnosis when analyzing CT images can enhance the practical utility of AI. | Pathology: Pulmonary Nodules,  No of the case: 50  Image modality: CT  Test observer: 4 radiologists.  (2–11 years  experience) | Machine Learning | 294 s without AI-Based CAD (time) Vs 274 s AI-Based CAD (time) | AI is significant for improving workflow efficiency and diagnostic precision in radiology practice. |
| Vijayalakshmi, S., et al.2021(28). India | To develop a machine learning model that predicts possible diseases based on patient symptoms, leveraging supervised learning techniques. | Population: Patients with various health conditions  Inputs: Patient symptom data.  Study Design: A computational study.  Measurements: Accuracy and model performance | Machine Learning | Supervised learning models can improve disease prediction accuracy, reducing reliance on traditional diagnostic methods. | This study highlights the potential of AI-driven healthcare, offering an automated and scalable approach for disease diagnosis. |
| Shin, H.J.,2023(29) | to examine the impact of AI on radiologists' reading times during the routine interpretation of CXRs | Pathology: Pulmonary Nodules,  No of the case: 18,680 Image modality: chest X-rays  Test observer: 11 radiologists. | Commercial AI software | 14.8 s without AI-Based CAD (time) Vs13.3 s AI-Based CAD (time) | AI can significantly reduce radiologists' reading times for chest X-rays, particularly when no abnormalities are detected. |
| Harinishree, M.2021(30). India | To analyze available datasets for training machine learning models and compare different ML techniques for breast cancer prediction. | Population: Women diagnosed with breast cancer.  Study Design: A comparative analysis | Machine Learning | The study provides a comprehensive comparison of different ML models, highlighting their effectiveness in breast cancer prediction. | This research underscores the potential of AI-driven diagnostics, offering computer-aided solutions to improve breast cancer detection. |
| Kim et al(31) | to evaluate the concordance rate of radiologists and Lunit for thoracic abnormalities in a multicenter health screening cohort. | Pathology: Pulmonary Nodules,  No of the case: 3,113 participants (1,157 men; mean age, 49 years)  Image modality: CXR | deep-learning algorithm | 19s without AI-Based Vs 14s AI-Based CAD (time) | The reading time slightly increased with the Lunit assistance. |
| Sakkos, D., et al.2021(32). United Kingdom | To automate cerebral palsy diagnosis using deep learning, improving interpretability through a visualization framework. | Population: Infants undergoing General Movements Assessment (GMA).  Inputs: RGB video sequences  Study Design: A comparative analysis | deep-learning algorithm | The deep learning framework outperformed previous pose-based techniques, demonstrating higher consistency and robustness in cerebral palsy classification. | This study highlights the potential of AI-driven diagnostics, offering interpretable and automated solutions for early cerebral palsy detection. |
| Basel Yacoub et al(33), 2022 | To assess the impact of an AI platform on radiologists' chest CT interpretation times in a real-world clinical setting. | Pathology: Pulmonary Nodules,  No of the case: 390 patients (204 women, 186 men; mean age, 62.8 ± 13.3 [SD] years)  Image modality: CT | a commercial AI software | 344 ± 129 seconds without AI-Based Vs 289 ± 89 seconds AI-Based CAD (time) | Integrating the AI support platform into the clinical workflow improved radiologist efficiency. |
| Baltruschat et al(34), 2021 German | To assess if AI-driven smart worklist prioritization can enhance radiology workflow efficiency and decrease report turnaround times (RTATs) for critical findings in chest radiographs (CXRs). | Pathology: Pulmonary Nodules,  No of the case: 10,000 chest X-rays Image modality: CXR | Artificial intelligence | 35.6 min without AI-Based Vs 80.1 min AI-Based CAD (time) | AI-driven smart worklist prioritization reduces the average RTAT for critical findings in CXRs. |
| Chauhan, T., et al, 2021. (35). India | To examine the effectiveness of supervised and unsupervised machine learning algorithms in diagnosing diabetes. | Population: Patients diagnosed with or at risk of diabetes  Study Design: A survey-based review analysing.  Measurements: prediction accuracy | Decision tree-based algorithms | K-Means and SVM demonstrated high accuracy in diagnosing diabetes, showcasing the effectiveness of hybrid ML approaches. | This study highlights the growing role of AI in healthcare, offering data-driven solutions for early diabetes detection. |
| Brown et al(36), 2018. | examines how a commercial chest CT -CAD system is integrated into the radiology reporting workflow and assesses its impact on radiologist efficiency. | Pathology: Pulmonary Nodules,  No of the case: 40 patients  Image modality: CT CAD  Test observer: 3 radiologists. | A commercial chest CT CAD software | reduced reading times by 7%–44% | Automated repopulations of a report with nodule and aorta measurements saved significant time compared to manual methods. |
| Silva et al (37)2018. | To compare CAD and visual reading for detecting subsolid nodules (SSNs) in volumetric LDCT scans for lung cancer screening. | Pathology: Pulmonary Nodules,  No of the case: 2303 patients  Image modality: CT CAD  Test observer: 2 experienced thoracic radiologists. | visual detection (VD) and management of SSN | Radiologists noted longer reading times due to false-positive findings | CAD systems assist in nodule detection but need visual confirmation to reduce false positives |
| Fersberge et al(38), 2017. | To evaluate radiologists' time variations in reading different procedure types. | Pathology: Pulmonary Nodules,  No of the case: 2303 patients  Image modality: CT CAD  Test observer: 2 experienced thoracic radiologists. | PACS usage logs | Similar baseline reading times of 366s and 370s, | workload distribution should consider radiologists' past performance as rankings vary across different procedure types. |
| Frank Diamond, Jul 17, 2023(39). | To explore how HealthCare Services (HCS) utilizes artificial intelligence (AI) to expedite the prior authorization process in healthcare. | Population: healthcare providers, patients, and insurance companies | Artificial intelligence | The implementation of AI in prior authorization reduced processing times, enhance accuracy in decision-making, and improve overall patient experience.  Health care service corporation (HCSC) implemented AI and observed a 1400 times faster processing rate for PA requests in 2022. | The use of AI in prior authorization can lead to increased efficiency in healthcare operations, reduced administrative burden on staff, and potentially better health outcomes for patients due to quicker access to necessary treatments. |
| Alam A, Prybutok VR, 2023(40). USA | To develop and implement responsible AI approaches to accurately predict health insurance claims in the USA using machine learning algorithms | Health insurance claim data from various demographics across the USA, including factors such as age, gender, health status, and previous claim history.  dataset consists of 1,340 rows and 11 columns, 10 of which are features, and 1 target variable of the  dataset for better understanding | Machine learning algorithm | XGBoost and RF models, achieving R-squared values of 79% and 77% respectively, outperformed other algorithms in forecasting health insurance claims, leading to improved risk assessment and resource allocation for insurance providers. | Implementation of this model can enhance the efficiency and fairness of health insurance processes, reduce fraudulent claims, and ultimately improve the overall quality of healthcare provision by enabling insurers to offer more tailored and equitable policies. |
| Michelle M. Mello, 2024(41) | investigates the potential benefits and risks of using AI in Medicare Advantage plans for coverage decisions, focusing on the potential for algorithmic bias and lack of transparency | Case studies: Analysing specific examples of AI-driven denials in Medicare Advantage plans. - Legal analysis: Examining relevant legal precedents and regulations governing AI in healthcare. | AI algorithm | AI tools used in Medicare Advantage plans have led to concerns about algorithmic bias, lack of transparency, and potential harm to patients.  - The "black box" nature of AI algorithms makes it difficult for patients to understand why their coverage was denied and to challenge those decisions.  - The study highlights the need for stronger federal regulations to govern the use of AI in health insurance coverage decisions, including transparency requirements and robust review processes. | - The study calls for careful consideration of the ethical and regulatory implications of using AI in healthcare, particularly in insurance coverage decisions. - It emphasizes the need for transparency, accountability, and human oversight to ensure that AI is used responsibly and does not perpetuate existing inequalities. - The study suggests that AI can be a valuable tool in healthcare, but only if used ethically and responsibly, with a focus on patient well-being and equity. |
| Dan Lodder, 2023(42). | to demonstrate how AI and machine learning platforms can be used to optimize revenue cycle management in oncology practices, ultimately reducing administrative burdens and improving the overall efficiency of reimbursement processes. | -Case studies , and Expert analysis  - oncology practices and the challenges they face in navigating the complex reimbursement landscape  -costs associated with billing and insurance | AI and machine learning algorithm | AI and machine learning platforms can be used to analyze vast amounts of healthcare financial data, identifying patterns and predicting potential claim denials before they are submitted. - By leveraging AI, oncology practices can significantly improve their clean claim rate, reducing the need for repeated submissions and appeals. - This improved efficiency can lead to increased reimbursement for oncology treatments, ultimately reducing the financial burden on patients. | - AI has the potential to revolutionize the way oncology practices manage their revenue cycles, leading to significant cost savings and improved efficiency. - By reducing administrative burdens, AI allows healthcare providers to focus more on patient care, ultimately improving the quality of care for cancer patients. - the importance of embracing new technologies like AI to address the challenges of healthcare reimbursement and ensure that patients have access to the treatments they need. |
| Fumiko Chino(43), 2023 | Explore the patient experience with prior authorization for cancer care. | Sample size: 178 patients  Study design: cross-sectional, anonymous survey | Machine Learning | PA caused delays, anxiety, and burden with most rating it poorly and experiencing decreased trust in healthcare. | It is crucial to ensure that polices intended to improve healthcare quality and efficiency do not inadvertently harm patients or create barriers to access to care. |
| Nathan YY , 2019 (44) | To demonstrate how the current system creates significant delays potentially jeopardizing patient outcomes, and to process solutions that prioritize patient care. | Case study  Retrospective and comparatives analysis. | Proton beam technology | The prior authorization process, while intended to ensure appropriate use of resource, is often overly burdensome and delays treatment unnecessarily. | They advocate for a more patient-centred approach that prioritizes the clinical judgement of physicians and ensures timely access to PBT for those who can benefit from it. |
| Leslie A. Lenert, 2 February 2023(45). USA | To propose an alternative to the current rule-based approach to PA automation, which is often criticized for being rigid and lacking human-centric consideration. | -Modern approaches for accessing and exchanging existing electronic health data.  -AI methods tailored to reflect the judgments of expert panels.  -Few shot learning approaches to prevent bias. | AI algorithm | AI can enhance the quality of oncology care by incorporating national clinical guidelines and developing specialty-oriented tools, leading to more informed and consistent decision-making, resulting in more accurate and efficient treatment plans and improved patient outcomes |  |
| Bingham B, 2022(46), USA. | To determine the practice-level and national cost burden of treatment-related prior authorization for academic radiation oncology practice. | -Developed TDABC process maps.  - Survey from the 2017 & 2018 American Society for Radiation Oncology Workforce  - Annual PA cost estimate | Time driven activity-based costing (TDABC) | Pre-authorization (PA) process for radiation treatment-related services imposes a substantial financial strain on healthcare institutions, costing an estimated $500,000 annually per institution, with the majority of costs related to approved treatments | The need for a comprehensive evaluation of PA process and the implementation of PA processes and the implementation of strategies to mitigate the associated costs and administrative burden. |
| Williams E, et al(47). 2023. | To develop a data harmonization pipeline for clinical datasets that utilizes the fast healthcare interoperability resources standard. | Sample size: 40, 000 patient.  Location: ICU | AI algorithm | FHIR-DHP's standardized and scalable approach to transforming raw clinical data into an AI-friendly format holds promise for unlocking the full potential of AI in streamlining authorization processes and revolutionizing healthcare by addressing data harmonization challenges | This research implies far-reaching, paving the way for a future where AI plays an increasingly important role in improving patient care and advancing medical knowledge. |
| Hyun Kim(48), etal. 2021. | To assess the perspectives of radiation and medical oncology trainees on the prior authorization process and its effects on their training and patient care. | Sample size: 1505 trains  Study: national survey  authorization process was assessed using Likert scales, free response question, and multiple response selection. | Machine Learning | most trainees 71% of radiation and medical oncology trainees reporting a concern for declining quality of patient care due to the prior authorization process | The trainees’ concern about the prior authorization process highlights the need for systemic changes to improve the efficiency and effectiveness of this process. |
| Michael Chernew(49), 2021. | To determine the proportion of total healthcare spending that goes towards administrative activities and analyse the specific types of administrative activities that contribute most significantly to the cost. | Analysing using micro-costing techniques.  Study design: survey among physicians | Health information technology | AI-driven insurance approvals can streamline processes, automate tasks, and potentially reduce administrative costs by up to 30%, allowing providers to focus more on patient care | The costs have a substantial impact on overall cost of healthcare, access to care, and the efficiency of the system |
| Kim et al.  (2018)(50) Korea, | To investigate the status and trends in post-mastectomy breast reconstruction in Korea. | Retrospective observational study design  Sample size: 31,155 mastectomy cases included 7,088 breast reconstruction cases. | Big data technology | Leveraging the Health Insurance Review and Assessment Services (HIRA) big data hub for breast reconstruction statistics yielded more accurate and objective research outcomes compared to traditional survey-based methods, providing a robust foundation for future studies. | The study highlights the positive impact of national health insurance coverage on increasing access to breast reconstruction, while also revealing evolving preferences for different reconstruction methods. These findings have important implications for healthcare policy, research, and clinical practice, informing the development of more effective and equitable breast reconstruction services in Korea and beyond. |
| Zwerwer etal(51), 2024. Germanye. | To analyse COVID-19 hospitalization costs for non-ICU and ICU patients | Sample: 598 non-ICU patients and 510 ICU patients diagnosed with COVID-19 Location: Frankfurt University hospital. | Mechanical ventilation | median hospitalization costs of €3,010 for non-ICU patients and significantly higher costs for ICU patients: €5,887 for those without mechanical ventilation (MV) and €21,536 for those requiring MV. Each additional day in the general ward cost around €414.20, while ICU costs per day were €927.45 without MV and €2,224.84 with MV. | The study suggests that strategies to shorten ICU stays could reduce overall costs, providing valuable insights for policymakers to develop strategies for managing the financial burden of COVID-19, including resource allocation, insurance coverage, and long-term care planning. |
| Dominique J. Monlezun, etal. 2022(52). USA | To optimize clinical effectiveness, cost efficiency, and health equity (for an optimal sustainable net benefit of patients and populations within the context of modern healthcare systems). | Model: CACOS Predictive Model Creation, Validation, and Calibration.  Study population: 122, 600 sample | Artificial intelligence | The cost-effectiveness of LHC was  $915.82 spent to avert one additional death.  -The net benefit was $59.72 billion. | This AI-guided analysis provides novel insights into LHC mortality reductions in cardio-oncology cardiac arrests and associated disparities. It also presents the first known clinical predictive model (CACOS) for cardiac arrest in active cancer. By utilizing a nationally representative dataset, the study emphasizes the need to reduce disparities in cardiac arrest prevalence, treatment, and outcomes based on factors such as race, income, insurance, and region. |
| [John R. Fischer](https://www.dotmed.com/news/search.html?search_author=264693&key=John+R.+Fischer), 2023 (53).  USA | To compare three AI models against human schedulers in predicting surgical case lengths | Sample size: 33,815 cases.  Setting: outpatient and inpatient  Variable: Over time frequency and overtime cost. | Machine learning (ML) | -13% increase in prediction accuracy for the AI models.  -Accuracy translated to reduced overtime hours, resulting in approximately $79,000 in savings over four months.  - The AI models improved prediction by 3.4% within a 20% margin of error and decreased under-prediction by 4.3%, | highlighting the potential of AI to enhance operating room scheduling efficiency, reduce overtime costs, and generate significant cost savings |
| [Natasha Rozario](https://pubmed.ncbi.nlm.nih.gov/?term=Rozario+N&cauthor_id=33180692), 2020(54). Canada. | To explore how AI can optimize operating room booking times, leading to a reduction in nursing overtime. | 10,553 cases / three years.  Variable:  Overtime frequency,  Undertime frequency,  Overtime minutes used  Overtime cost,  OR minutes used, | Machine Learning | -The results showed a theoretical cost savings of $469,000 over three years by reducing nursing overtime by 21%. | While this study doesn't directly compare traditional methods to AI, it highlights AI's potential to impact overtime costs significantly. |
| Lena Zaubitzer (55) Germany. | To assess time management in the OR of ENT | Analysis: a retrospective analysis  Sample size: 1809 operations under general anaesthesia (22 different types) by 31  surgeons (12 specialists (S) and 19 residents (R)).  Incision: suture and preparation and post-processing times  of 10 specialists and 17 residents | Machine learning | Peer Well’s AI-powered PreHab platform cuts surgery costs by $1,215 per patient, enabling shorter hospital stays, fewer home care needs, and dramatically reduced skilled nursing facility discharges without in-person clinicians. | Peer Well’s AI-powered PreHab platform dramatically improves patient outcomes and reduces healthcare costs, resulting in shorter hospital stays and fewer post-surgical complications. |
| Stromblad et al.(56), | To assess accuracy and real-world outcomes to predict surgical case duration. | Studies included:  - scheduled more than 1 day before surgery.  -time: April 7, 2018, and June 25, 2018, were included.  - 29 strata (11 gynaecological surgeons at two campuses and seven colorectal surgeons at a single campus) to ensure an equal chance of selection for each surgeon and each campus. | Machine learning | The 8.3% decrease in significant prediction errors (MAE > 60 minutes) translates to fewer instances of case cancellations or extended operating room (OR) hours. | A significant reduction in prediction errors leads to shorter patient wait times and fewer scheduling disruptions, key factors in minimizing overtime. |
| Tuwatananurak et al.(57) | To estimate the improvement of surgical case duration. | Sample size: 1059 surgical cases.  Variables:  Overtime minutes used  Overtime cost,  OR minutes used,  Demographic | Machine learning | AI 70% reduction in scheduling inaccuracy, representing considerable savings considering OR costs ranging from $22 to $133 per minute. | ML-driven surgical scheduling significantly reduces overtime expenses. |
| Tomas Ramola, Germany(58), 2022. | To assess the utilization of AI | Sample size: 4,225 surgeries scheduled.  Study period: November 2021 and May 2022.  Variables: Overtime minutes used overtime cost. | Machine learning | -AI predictions for surgery durations were 30% more accurate than traditional methods.  -The AI's precision allowed for an 18.4% improvement in operating room (OR) scheduling, resulting in a 39% increase in correctly planned surgeries. Additionally, surgeries that exceeded the expected duration were completed 7.3% faster on average, and the accuracy of estimating shorter surgeries improved by 30.3%. | Even with the hospital's strong historical data, the impressive results suggest that facilities with less rigorous planning could experience even more significant cost savings. |
| Zhang et al.(59), | to reduce the high costs associated with surgical cancellations by identifying high-risk procedures. | Study period: January 2013- December 2024.  Sample size: 5,125 cases, of which 810 were cancelled  (positive) and 4,315 were not | Machine learning models | finding random forest to be the most accurate (0.8578 accuracy, 0.7199 AUC). | While demonstrating high specificity and negative predictive value, the study highlighted the need for improved sensitivity and positive predictive value to minimize cancellation costs further. |
| Luo et al(60) | To assess the high cost of surgical cancellations | Employing random forest, SVM, and XGBoost, their models effectively  Wilcoxon and chi-square tests were used for predictor selection. | Machine learning (ML) | A random forest model achieved an AUC > 0.6 in identifying high-risk cancellations, | highlighting the potential for significant cost savings through improved prediction and resource allocation. |
| Karim, S.A., et al. (61), 2023 | To determine the factors associated with telehealth adoption by rural hospitals and compare the financial performance of telehealth adopters and non-adopters. | -A longitudinal retrospective cohort design, analysing data from a panel dataset from 2009 to 2019.  -Logistic regression model.  - Pooled ordinary least squares regression with robust standard errors | ----------- | The study found that rural hospitals with telehealth technology had significantly higher operating margins compared to those without telehealth technology. This suggests a positive association between telehealth adoption and financial performance. | The study's findings highlight the potential of telehealth to improve the financial health of rural hospitals. Investing in telehealth technology could be a strategic move for rural hospitals struggling to stay afloat. However, the study also acknowledges that the financial benefits of telehealth are not always realized and that factors such as limited reimbursement, low volumes, and insufficient broadband can hinder its economic viability. |
| Lie Huang(62) | To assess an AI-assisted anaesthesia system to reduce emergency and recovery times following endoscopic surgery. | Sample size: 154 patients (76 in the AI group, 78 in the control group).  AI Training: An AI model was trained to recognize surgical phases from the videos using a deep-learning approach.  Model Evaluation: comparing its predictions to surgeon-annotated references. | machine learning algorithms, | Surgical complexity, measured by extended total surgical duration, bleeding, and complications, was strongly associated with the intraoperative surgical process, especially in the beginning phases (AUC 0.913).  - The AI model could recognize surgical phases from video with 87% accuracy.  - The AI model could determine intraoperative surgical complexity by calculating the duration of the beginning phases (AUC 0.859). | Surgical complexity can be predicted by the surgical process, particularly in the early stages.  - AI-based models can be used to automate the evaluation of surgical complexity.  - This technology could improve patient safety and surgical outcomes by enabling surgeons to anticipate and manage complex cases better. |
| Song's(63) | demonstrated the successful prediction of post-anaesthesia hypotension using AI models. | Sample size: 13,323 patients | machine learning algorithms, | This predictive capability is crucial for patient safety and a smoother aesthetic experience, potentially reducing surgical stress. | The study emphasizes the need for further research and development to address the challenges associated with AI in anaesthesia. It suggests that careful consideration of ethical, legal, and practical issues is crucial for successful AI implementation in this field.  The study highlights the potential of AI to improve anaesthesia practices significantly but also emphasizes the importance of responsible development and implementation to ensure patient safety and ethical use of technology. |
| KAJ et al(64). | To develop an AI-based deformable image registration method that offers an accurate and rapid registration process. | Chest modality: Pulmonary CT | Convolutional  neural networks | AI-based deformable image registration method offers an accurate and rapid registration process without parameterization during testing or manually annotated data for training. | The AI-based deformable image registration method outlined in the finding presents a transformative impact on medical imaging by enhancing efficiency, accuracy, speed, and versatility while reducing manual Labor and the need for extensive parameterization. |
| Kanavati F(65), 2020. | To develop a classification model that can accurately identify and classify different types of lung carcinoma using deep learning techniques. | No of the case: a dataset of 3,554 WSIs  Chest modality: Pulmonary CT | Deep learning | DL technology enhances precision and potentially improves the efficiency and outcomes of tumour boundary delineation procedures during surgery. | The significant impact of deep learning (DL) methodologies in real-time guidance, particularly in outlining tumour boundaries during surgical procedures. |
| Kanavati et al, 2006(66). | To Create a probabilistic neural network to predict the immediate postoperative prognosis of patients after resection by analysing numerous clinical and physiological factors. | No of the case: 141 patients  Chest modality: Pulmonary CT | CNN models | transforms lung cancer surgery by using DL to enhance precision, minimize tissue damage, and optimize tumour removal, surpassing human capabilities in real-time guidance. | Introduce a dynamic element into the surgical environment, providing real-time insights and support to healthcare professionals. |
| J. Pao et al, (67)2023 | To predict EGFR mutations from pathology images | No of the case: 2099 patients  Chest modality: pathology image. | Deep learning | AI acts as a real-time decision support tool, augmenting the surgeon's expertise with AI's analytical prowess. | As AI technology advances, we expect even more sophisticated applications to revolutionize surgical practice and enhance patient care. |
| Jiao et al(68).2022 | Developed an ML approach integrating preoperative and intraoperative data for continuous surgery duration prediction. | Total no. of patients’ records used: 69 018  Number of patients used for training/ testing:  59 926/9092 (92.5%/7.5% | Bayesian approach, modular artificial neural network | Performance of AI model(s) versus control: 20.3 min vs 37.0 min & 13.8 min Vs 37.0 min  AI model more accurate than control (statistically significant): P < 0.001 (for MANN versus Bayesian approach) | ML models play a crucial role in guiding operational decisions, surpassing classical scheduling methods and traditional statistical alternatives, potentially leading to cost savings in surgery by accurately predicting staff overtime and enhancing resource allocation. |
| *Ershad M,* et al(69). 2019 | To develop a framework for automatic stylistic behaviour recognition in robotic surgery, which could be implemented in near real-time. | Application: Evaluation of  technical skills in  robotic surgery  Number of participants: 10 | Support vector  Machine | The proposed dictionary learning method can assess stylistic behaviour  performance in near real-time with improved accuracy compared to using PCA features or raw data. | This real-time feedback system could enable trainees to adjust their movements during surgical tasks, potentially resulting in improved performance and decreased complication rates in real surgical scenarios. |
| *Huang et al.(70) 2017* | Created a low-cost, automated OR usage system and analysed data from various operating rooms. | -Number of cases: 1003 cases  -Smart OR automatically and reliably captures data | Machine learning | the average turnover time was 36 minutes, with 38% of cases achieving the target of 30 minutes or less. | The introduction of the Smart OR system, which automates data capture and provides real-time monitoring, allows OR teams to concentrate on improving efficiency without the need for manual data entry. |
| *Bendixen et al(71). 2016* | To compare pain and quality of life in VATS VS open surgery for lung cancer. | -Number of cases: 206  -Assessed longitudinally by logistic regression  - modality: pathology. | Video-assisted thoracoscopic surgery (VATS) | VATS patients experienced notably reduced pain levels and improved quality of life within the first-year post-surgery. | The integration of AI in minimally invasive surgeries like VATS holds promise in enhancing surgical precision, optimizing patient outcomes, and supporting surgeons in decision-making processes, ultimately benefiting individuals undergoing lobectomy for stage I non-small-cell lung cancer. |
| Connor, C.W., (72) | explored the use of AI for automated propofol dosing during surgery. | A longitudinal retrospective cohort design, analysing data from a panel dataset spanning from 2009 to 2019.  -Logistic regression model.  - Pooled ordinary least squares regression with robust standard errors | machine-learning algorithm | This suggests that AI could free up anaesthesiologists' time for other tasks, reducing their cognitive workload. | AI and ML have the potential to significantly improve anthologies practices, leading to enhanced patient safety, improved clinical outcomes, and more efficient and effective care delivery. |
| Xu, C., et al.,(73). | demonstrated the use of AI to improve anaesthesia quality control during gastrointestinal endoscopy. | Total no. of patients’ records used: 69 018  Number of patients used for training/ testing:  59 926/9092 (92.5%/7.5% | Machine learning | The AI-powered systems could help anaesthetists optimize sedation levels, leading to shorter recovery times and increased patient satisfaction. | This finding highlights the potential of AI for personalized anaesthesia management based on individual patient responses. |
| De Vos et al. (2022)(74). Holand | Assessed cost-effectiveness of personalized care (PC) compared to standard care in the Dutch ICU from a societal perspective. | Model type: Markov model  Time horizon cycle length: 1 year, 1 day  Intervention: AI decision support tool  for ICU discharge  decision-making  Perspective: societal  Population: 1000 adult Dutch patients admitted in the ICU.  Care pathway phase: diagnostic | Machine learning  AI technology: clinical decision support | EUR 18,507 per QALY gained  vs. standard care  Intervention cost effective. | machine learning prediction model Pacmed Critical (PC) could be a valuable investment for Dutch ICUs, offering cost-effectiveness and potential improvement patient outcomes through reduced ICU length of stay. |
| Lee et al (2019)(75), USA | total joint replacement (TJR) readmissions prediction and cost-effective intervention. | Care pathway: intervention and treatment  Population: Adult patients (men and  women) with total joint  replacements  Intervention: AI predictive model for  readmission risk  Time horizon: 90 days  Model type: decision tree  Sample size: 1200 patient. | ML prediction model with RUS Boost for total joint replacement readmission risk  AI technology: pattern recognition | Total costs $651 490,  $1 994 654 and $963 550  Lowest total  cost for high-risk patients  with home service | AI improve patient outcomes, reduce costs, and enhance healthcare quality after total joint replacement surgery by predicting readmissions and implementing cost-effective interventions for high-risk patients |
| Eigner et al, 2020(76). Asia Pasfic. | To predict re admission risk and optimize discharge time | Population: Adult patients hospitalized for a variety of major surgeries  Care pathway: follow up  Intervention: Decision algorithm for  optimal time of patient  discharge  Time horizon: 28 day | AI Prediction modelling for readmission risk | machine learning can predict readmission risk and help hospitals determine the best time to discharge patients, balancing safety, quality, cost, and resources. It found that extending the stay for certain patient groups can save money by reducing readmissions. This data-driven approach helps doctors and hospital managers make better decisions, leading to improved patient outcomes, lower costs, and better healthcare quality. | |
| Oskar Ericson et al(77), 2022. Sweden | Sepsis prediction algorism: cost and effectiveness in ICU | Population: Adult  Model: Health economics  Sample: 5860 patients at ICU  Location: Hospital | Machine learning NAVOY® Sepsis algorithm | Potential cost saving of €76 per patient, with significant reductions in ICU stays leading to savings of €1009 per ICU patient. The algorithm emerged as a dominant and cost-effective treatment option in stochastic scenario analysis, staying well below the threshold of €20,000 per QULY | The transformative impact of technology on healthcare, presenting NAVOY® Sepsis as a tool that not only eases the financial burden on healthcare systems but also serves as a powerful lifesaving solution for sepsis management. |
| Jacob Calvert(78), 2017. USA | Sepsis prediction algorism driven biomarker: financial and mortality impact of Insight | Location: inpatient setting  Sample: individual ICU with 50 beds Model: Health economics | Machine learning algorithm-driven sepsis prediction | This translates into tangible benefits for healthcare facilities, including saving an estimated 75 lives annually in a 50-bed ICU and reducing sepsis-related costs by $560,000. | findings paint a compelling picture of InSight as a life-saving innovation with the potential to significantly impact the fight against sepsis, saving lives and reducing healthcare costs, offering hope for a future where sepsis is no longer a silent killer. |
| Leslie R. Zwerwer etal(79), Germaney | The role of AI for the treatment of mechanically  ventilated ICU patients | Generic health economic  Model  Sample: mechanically ventilated patients  Location: hospital | AI systems | This model empowers investors and innovators to determine the financial viability of implementing specific AI systems even before their clinical impact is fully established, allowing for informed decision-making across diverse clinical scenarios | The finding represents a significant step forward in integrating AI into healthcare, empowering investors, innovators, and policymakers to make informed decisions that drive the development and adoption of AI technologies that benefit both patients and healthcare systems. |
| Aanesen et al  (2010)(80), Norway | Examine the role  of new technology maintaining an old working  procedure | Sample size: 9 physicians and 10 hospital departments.  Intervention: Discharge summary created  electronically and sent  electronically  Comparator: Paper-based discharge | electronic discharge  communication tools | Electronic message exchange in hospitals and primary care saves money. Over 5 years, the net savings are €31.1 million. Over 10 years, the savings reach €24.6 million. | Decision-makers should prioritize cost-effective options when choosing electronic discharge tools, especially during challenging economic times in healthcare. |
| Mourad et al (2011)(81), USA. | To assess business case for the  implementation of an electronic  discharge summary | Sample size: 600 bed quaternaries  care academic  institution.  Intervention: Separate software  tracks signatures and  automatically triggers  dissemination  comparator: Orally dictated discharge notes | Software  tracks signatures | Current discharge practices cost $496,400 annually, with billing delays adding $107,000 to $215,000 in costs. | Investing in e-discharge provides immediate advantages for patients, system efficiency, quality improvements, and financial returns. |
| Sales, B.,2011 (82) Brazil | To develop a **collaborative virtual environment** for medical training | Population: Medical students and professionals  Study Design: A computational study  Measurements: Usability, effectiveness, and engagement. | Machine learning | The CyberMed collaboration module enhances remote medical training, allowing professionals to simulate real-world procedures in a shared virtual space. | The potential of VR-based medical education, offering interactive and accessible training solutions for geographically dispersed learners. |
| Tsai, M.-D., et al.2011, (83) Taiwan | To develop a soft tissue manipulation method integrated into a virtual reality surgical simulator for facial contouring surgery | **Population: Patients undergoing facial contouring surgery**  Study Design: A computational study  simulation accuracy, visualization quality, and usability | Machine learning | The integrated simulator provides high-quality illustrations for various facial contouring surgery modalities, improving surgical planning and training. | This study highlights the potential of VR-based medical simulations, offering realistic and interactive tools for facial surgery planning. |
| Cecil, J. and M. Pirela-Cruz.2013 (84). Netherland & UK | To develop an information model using Enterprise Modelling Language (eEML) | **Population: Medical residents**  **Study Design: A computational study**  **Measurements: usability, effectiveness, and engagement** | Machine learning | The eEML-based information model provides a structured approach to virtual surgical training, improving skill development and procedural understanding. | This study highlights the potential of VR and haptic interfaces in medical education, offering interactive and accessible training solutions for orthopaedic surgery. |
| Paranjape, K. et al.2019 (85). UK | To analyse the current state of medical education and propose a framework for integrating AI into the curriculum. | Population: Medical professionals and students.  **Study Design: A qualitative analysis**  **Measurements: cost-effectiveness, quality improvement, accessibility, and ethical concerns** | AI | AI integration in medical education can enhance clinical decision-making, but challenges such as transparency and liability must be addressed. | This study highlights the importance of AI literacy for future healthcare professionals, advocating for seamless AI integration across medical curricula. |
| Torner, J., et al. (86). Spain | To develop an open-source VR environment suitable for multiple biomedical and healthcare applications, | Population: Pilot subjects tested the VR system.  Study Design: A computational study  Measurements: evaluates usability, effectiveness, and adaptability of the VR system. | Machine learning | The VR environment successfully supports motor rehabilitation and real-time neurofeedback, demonstrating high adaptability and usability. | This study highlights the potential of VR in biomedical applications, offering immersive and interactive solutions for healthcare and rehabilitation. |
| Gupta, A., et al., 2019(87). | To design a cyber-human systems-based simulator framework for training orthopaedic surgery residents | Population: Medical residents special  Study Design: A computational study  Measurements: The study evaluates usability, effectiveness, and engagement | Machine learning | The ICSE-based simulator framework provides a structured approach to virtual surgical training, improving skill development and procedural understanding. | This study highlights the potential of VR and haptic interfaces in medical education, offering interactive and accessible training solutions for orthopedic surgery. |
| Wiljer, D. and Z. Hakim.2019 (88) Canada | To analyze the current state of AI adoption in healthcare and propose a framework for integrating AI education | Population: Healthcare professionals,  Study Design: A qualitative analysis  measurements: AI literacy, data governance, algorithm readiness, and clinical impact. | AI | AI integration in healthcare education can enhance clinical decision-making, but challenges such as data access and algorithm transparency must be addressed. | This study highlights the importance of AI literacy for healthcare professionals, advocating for structured AI education and training programs. |
| Deist, T.M., et al. 2020 (89) Amsterdam, Cardiff, Maastricht, Manchester, Nijmegen, Rome, Rotterdam, Shanghai | To develop a privacy-preserving infrastructure for distributed healthcare data analysis | **Population:** Lung cancer patients  Sample Size: 23,203 patients  Measurements: logistic regression model. | Machine learning algorithms | The PHT infrastructure successfully enables privacy-preserving distributed learning, overcoming data-sharing barriers while supporting global evidence-based medicine. | This study highlights the potential of federated learning in healthcare, ensuring secure and scalable AI-driven medical research. |
| Jumelle, A.K.L., et al.2014 (90) | To identify and address ethical concerns in the adoption of Future Internet technologies for healthcare. | **Population:** Healthcare professionals, developers, and policymakers  **Inputs: Ethical considerations Study Design: A policy analysis**  **Measurements: evaluates ethical impact assessment, regulatory needs, and strategies for ethical compliance.** | Machine learning algorithms | The study proposes an overarching e-Health ethical framework, an ethical impact assessment, and an ethical matrix to guide decision-making. | This research highlights the importance of ethical governance in digital healthcare, ensuring privacy, security, and equitable access. |
| Antoniou, Z.C., et al.,2017 (91). UK | Develop an adaptive video encoding framework for mobile health video communication, ensuring real-time optimization for clinical diagnosis. | **Population: Evaluated on ultrasound videos**  **Study Design: Multi-objective optimization framework**  **Sample Size: Ten ultrasound videos.**  **Measurements: Structural similarity quality, bitrate demand, encoding frame rate, and real-time adaptation performance.** | Machine learning | The framework demonstrated significant improvements over static encoding methods, achieving enhanced video quality, lower bitrate demands, and real-time adaptation capabilities. | The approach is generalizable to other medical video modalities. |
| Jaiman, V. and V. Urovi.2020.(92) | Develop a blockchain-based consent model to enhance secure and accountable sharing of electronic health records (EHRs). | **Population: Individuals sharing health data and data requesters**  **Study Design: Experimental evaluation**  **Measurements: Efficiency, adaptability of the dynamic consent model, and performance evaluation** | Machine learning | The proposed model ensures flexible, privacy-preserving decision-making, allowing individuals to dynamically control access to their health data. | The results indicate that the model is efficient and aligns with GDPR compliance, making it a viable approach for secure EHR data-sharing. |

**Reference**

1. Bernardini M, Romeo L, Frontoni E, Amini M-R. A semi-supervised multi-task learning approach for predicting short-term kidney disease evolution. IEEE Journal of Biomedical and Health Informatics. 2021;25(10):3983–94.

2. Yu H, Zhou Z. Optimization of IoT-based artificial intelligence assisted telemedicine health analysis system. IEEE access. 2021;9:85034–48.

3. Dhieb N, Ghazzai H, Besbes H, Massoud Y. A secure ai-driven architecture for automated insurance systems: Fraud detection and risk measurement. IEEE Access. 2020;8:58546–58.

4. Sqalli MT, Al-Thani D, editors. AI-supported health coaching model for patients with chronic diseases. 2019 16th International Symposium on Wireless Communication Systems (ISWCS); 2019: IEEE.

5. Zhou L, Li Z, Zhou J, Li H, Chen Y, Huang Y, et al. A rapid, accurate and machine-agnostic segmentation and quantification method for CT-based COVID-19 diagnosis. IEEE transactions on medical imaging. 2020;39(8):2638–52.

6. Dagan N, Elnekave E, Barda N, Bregman-Amitai O, Bar A, Orlovsky M, et al. Automated opportunistic osteoporotic fracture risk assessment using computed tomography scans to aid in FRAX underutilization. Nature medicine. 2020;26(1):77–82.

7. Sasubilli SM, Kumar A, Dutt V, editors. Machine learning implementation on medical domain to identify disease insights using TMS. 2020 International Conference on Advances in Computing and Communication Engineering (ICACCE); 2020: IEEE.

8. Kaur A, Garg R, Gupta P, editors. Challenges facing AI and Big data for Resource-poor Healthcare System. 2021 second international conference on electronics and sustainable communication systems (icesc); 2021: IEEE.

9. Thakkar BA, Hasan MI, Desai MA, editors. Health care decision support system for swine flu prediction using naïve bayes classifier. 2010 International Conference on Advances in Recent Technologies in Communication and Computing; 2010: IEEE.

10. Moein M, Davarpanah M, Montazeri MA, Ataei M, editors. Classifying ear disorders using support vector machines. 2010 second international conference on computational intelligence and natural computing; 2010: IEEE.

11. O’neill TJ, Xi Y, Stehel E, Browning T, Ng YS, Baker C, et al. Active reprioritization of the reading worklist using artificial intelligence has a beneficial effect on the turnaround time for interpretation of head CT with intracranial hemorrhage. Radiology: Artificial Intelligence. 2020;3(2):e200024.

12. Kim JH, Kim JY, Kim GH, Kang D, Kim IJ, Seo J, et al. Clinical validation of a deep learning algorithm for detection of pneumonia on chest radiographs in emergency department patients with acute febrile respiratory illness. Journal of Clinical Medicine. 2020;9(6):1981.

13. Liu P-Y, Tsai Y-S, Chen P-L, Tsai H-P, Hsu L-W, Wang C-S, et al. Application of an artificial intelligence trilogy to accelerate processing of suspected patients with SARS-CoV-2 at a smart quarantine station: Observational study. Journal of medical Internet research. 2020;22(10):e19878.

14. Osareh A, Shadgar B, editors. Machine learning techniques to diagnose breast cancer. 2010 5th international symposium on health informatics and bioinformatics; 2010: IEEE.

15. Zhang D, Liu X, Shao M, Sun Y, Lian Q, Zhang H. The value of artificial intelligence and imaging diagnosis in the fight against COVID-19. Personal and ubiquitous computing. 2023;27(3):783–92.

16. Ladgham A, Torkhani G, Sakly A, Mtibaa A, editors. Modified support vector machines for MR brain images recognition. 2013 International Conference on Control, Decision and Information Technologies (CoDIT); 2013: IEEE.

17. Kozuka T, Matsukubo Y, Kadoba T, Oda T, Suzuki A, Hyodo T, et al. Efficiency of a computer-aided diagnosis (CAD) system with deep learning in detection of pulmonary nodules on 1-mm-thick images of computed tomography. Japanese Journal of Radiology. 2020;38:1052–61.

18. Razaak M, Martini MG, Savino K. A study on quality assessment for medical ultrasound video compressed via HEVC. IEEE Journal of biomedical and health informatics. 2014;18(5):1552–9.

19. Liu K, Li Q, Ma J, Zhou Z, Sun M, Deng Y, et al. Evaluating a fully automated pulmonary nodule detection approach and its impact on radiologist performance. Radiology: Artificial Intelligence. 2019;1(3):e180084.

20. Gayathri B, Sumathi C, editors. Mamdani fuzzy inference system for breast cancer risk detection. 2015 IEEE international conference on computational intelligence and computing research (ICCIC); 2015: IEEE.

21. Martini K, Blüthgen C, Eberhard M, Schönenberger A, De Martini I, Huber F, et al. Impact of vessel suppressed-CT on diagnostic accuracy in detection of pulmonary metastasis and reading time. Academic radiology. 2021;28(7):988–94.

22. Zhou Y, Xu J, Liu Q, Li C, Liu Z, Wang M, et al. A radiomics approach with CNN for shear-wave elastography breast tumor classification. IEEE Transactions on Biomedical Engineering. 2018;65(9):1935–42.

23. Nam JG, Kim M, Park J, Hwang EJ, Lee JH, Hong JH, et al. Development and validation of a deep learning algorithm detecting 10 common abnormalities on chest radiographs. European Respiratory Journal. 2021;57(5).

24. Saxena S, Prasad S, editors. Machine learning based sensitivity analysis for the applications in the prediction and detection of cancer disease. 2019 IEEE International Conference on Distributed Computing, VLSI, Electrical Circuits and Robotics (DISCOVER); 2019: IEEE.

25. Sung J, Park S, Lee SM, Bae W, Park B, Jung E, et al. Added value of deep learning–based detection system for multiple major findings on chest radiographs: a randomized crossover study. Radiology. 2021;299(2):450–9.

26. Zhang Y, Wei Y, Wu Q, Zhao P, Niu S, Huang J, et al. Collaborative unsupervised domain adaptation for medical image diagnosis. IEEE Transactions on Image Processing. 2020;29:7834–44.

27. Beyer F, Zierott L, Fallenberg E, Juergens K, Stoeckel J, Heindel W, et al. Comparison of sensitivity and reading time for the use of computer-aided detection (CAD) of pulmonary nodules at MDCT as concurrent or second reader. European radiology. 2007;17:2941–7.

28. Vijayalakshmi S, Saini A, Srinivasan A, Singh NK, editors. Disease prediction over big data from healthcare institutions. 2021 International Conference on Advance Computing and Innovative Technologies in Engineering (ICACITE); 2021: IEEE.

29. Shin H, Han K, Ryu L, Kim E. The impact of artificial intelligence on the reading times of radiologists for chest radiographs. npj Digital Medicine, 6, 82. 2023.

30. Harinishree M, Aditya C, Sachin D, editors. Detection of breast cancer using machine learning algorithms–a survey. 2021 5th International Conference on Computing Methodologies and Communication (ICCMC); 2021: IEEE.

31. Kim EY, Kim YJ, Choi W-J, Jeon JS, Kim MY, Oh DH, et al. Concordance rate of radiologists and a commercialized deep-learning solution for chest X-ray: Real-world experience with a multicenter health screening cohort. PLoS One. 2022;17(2):e0264383.

32. Sakkos D, Mccay KD, Marcroft C, Embleton ND, Chattopadhyay S, Ho ES. Identification of abnormal movements in infants: A deep neural network for body part-based prediction of cerebral palsy. IEEE Access. 2021;9:94281–92.

33. Yacoub B, Varga-Szemes A, Schoepf UJ, Kabakus IM, Baruah D, Burt JR, et al. Impact of artificial intelligence assistance on chest CT interpretation times: a prospective randomized study. American Journal of Roentgenology. 2022;219(5):743–51.

34. Baltruschat I, Steinmeister L, Nickisch H, Saalbach A, Grass M, Adam G, et al. Smart chest X-ray worklist prioritization using artificial intelligence: a clinical workflow simulation. European radiology. 2021;31:3837–45.

35. Chauhan T, Rawat S, Malik S, Singh P, editors. Supervised and unsupervised machine learning based review on diabetes care. 2021 7th International Conference on Advanced Computing and Communication Systems (ICACCS); 2021: IEEE.

36. Brown M, Browning P, Wahi-Anwar MW, Murphy M, Delgado J, Greenspan H, et al. Integration of chest CT CAD into the clinical workflow and impact on radiologist efficiency. Academic radiology. 2019;26(5):626–31.

37. Silva M, Schaefer-Prokop CM, Jacobs C, Capretti G, Ciompi F, van Ginneken B, et al. Detection of subsolid nodules in lung cancer screening: complementary sensitivity of visual reading and computer-aided diagnosis. Investigative radiology. 2018;53(8):441–9.

38. Forsberg D, Rosipko B, Sunshine JL. Radiologists’ variation of time to read across different procedure types. Journal of digital imaging. 2017;30(1):86–94.

39. Diamond F. How HCSC is using AI to speed up prior authorization. Jul 17, 2023.

40. Alam A, Prybutok VR. Use of responsible artificial intelligence to predict health insurance claims in the USA using machine learning algorithms. Exploration of Digital Health Technologies. 2024;2(1):30–45.

41. Mello MM, Rose S, editors. Denial—Artificial Intelligence Tools and Health Insurance Coverage Decisions. JAMA Health Forum; 2024: American Medical Association.

42. Lodder D. The Future of Reimbursement: How AI Can Revolutionize Oncology Practices. Oncology LIVE. 2023:NA–NA.

43. Chino F, Baez A, Elkins IB, Aviki EM, Ghazal LV, Thom B. The patient experience of prior authorization for cancer care. JAMA Network Open. 2023;6(10):e2338182–e.

44. Nathan YY, Sio TT, Mohindra P, Regine WF, Miller RC, Mahajan A, et al. The insurance approval process for proton beam therapy must change: Prior authorization is crippling access to appropriate health care. International Journal of Radiation Oncology, Biology, Physics. 2019;104(4):737–9.

45. Lenert LA, Lane S, Wehbe R. Could an artificial intelligence approach to prior authorization be more human? Journal of the American Medical Informatics Association. 2023;30(5):989–94.

46. Bingham B, Chennupati S, Osmundson EC. Estimating the practice-level and national cost burden of treatment-related prior authorization for academic radiation oncology practices. JCO Oncology Practice. 2022;18(6):e974–e87.

47. Williams E, Kienast M, Medawar E, Reinelt J, Merola A, Klopfenstein SAI, et al. A standardized clinical data harmonization pipeline for scalable ai application deployment (fhir-dhp): Validation and usability study. JMIR Medical Informatics. 2023;11:e43847.

48. Kim H, Srivastava A, Gabani P, Kim E, Lee H, Pedersen KS. Oncology trainee perceptions of the prior authorization process: a national survey. Advances in Radiation Oncology. 2022;7(2):100861.

49. Chernew M, Mintz H. Administrative expenses in the US health care system: why so high? JAMA. 2021;326(17):1679–80.

50. Kim J-W, Lee J-H, Kim T-G, Kim Y-H, Chung KJ. Breast reconstruction statistics in Korea from the big data hub of the Health Insurance Review and Assessment service. Archives of Plastic Surgery. 2018;45(05):441–8.

51. Zwerwer LR, Kloka J, van der Pol S, Postma MJ, Zacharowski K, van Asselt AD, et al. Mechanical ventilation as a major driver of COVID-19 hospitalization costs: a costing study in a German setting. Health economics review. 2024;14(1):4.

52. Monlezun DJ, Sinyavskiy O, Peters N, Steigner L, Aksamit T, Girault MI, et al. Artificial intelligence-augmented propensity score, cost effectiveness and computational ethical analysis of cardiac arrest and active cancer with novel mortality predictive score. Medicina. 2022;58(8):1039.

53. por John R. Fischer. Providers see lower overtime costs when AI sets surgical schedule. Annals of Surgery Health care business new July 03, 2023.

54. Rozario D. Can machine learning optimize the efficiency of the operating room in the era of COVID-19? Canadian Journal of Surgery. 2020;63(6):E527.

55. Zaubitzer L, Affolter A, Büttner S, Ludwig S, Rotter N, Scherl C, et al. Time management in operating rooms—a cross-sectional study to evaluate estimated and objective durations of otorhinolaryngologic surgical procedures. HNO. 2022:1–9.

56. Strömblad CT, Baxter-King RG, Meisami A, Yee S-J, Levine MR, Ostrovsky A, et al. Effect of a predictive model on planned surgical duration accuracy, patient wait time, and use of presurgical resources: a randomized clinical trial. JAMA surgery. 2021;156(4):315–21.

57. Tuwatananurak JP, Zadeh S, Xu X, Vacanti JA, Fulton WR, Ehrenfeld JM, et al. Machine learning can improve estimation of surgical case duration: a pilot study. Journal of medical systems. 2019;43:1–7.

58. Ramolla DMT. Optimize OR utilization with artificial intelligence (AI). May 2022, .

59. Zhang F, Cui X, Gong R, Zhang C, Liao Z. Key Experimental Factors of Machine Learning‐Based Identification of Surgery Cancellations. Journal of Healthcare Engineering. 2021;2021(1):6247652.

60. Luo L, Zhang F, Yao Y, Gong R, Fu M, Xiao J. Machine learning for identification of surgeries with high risks of cancellation. Health Informatics Journal. 2020;26(1):141–55.

61. Karim SA, Tilford JM, Bogulski CA, Rabbani M, Hayes CJ, Eswaran H. Financial performance of rural hospitals persistently lacking or having telehealth technology. Journal of Hospital Management and Health Policy. 2023;7.

62. Huang L, Chen X, Liu W, Shih P-C, Bao J. Automatic surgery and anesthesia emergence Duration prediction using artificial neural Networks. Journal of Healthcare Engineering. 2022;2022(1):2921775.

63. Song B, Zhou M, Zhu J. Necessity and importance of developing AI in anesthesia from the perspective of clinical safety and information security. Medical Science Monitor: International Medical Journal of Experimental and Clinical Research. 2023;29:e938835–1.

64. Eppenhof KA, Pluim JP. Pulmonary CT registration through supervised learning with convolutional neural networks. IEEE transactions on medical imaging. 2018;38(5):1097–105.

65. Kanavati F, Toyokawa G, Momosaki S, Rambeau M, Kozuma Y, Shoji F, et al. Weakly-supervised learning for lung carcinoma classification using deep learning. Scientific reports. 2020;10(1):9297.

66. Esteva H, Marchevsky A, Nunez T, Luna C, Esteva M. Neural networks as a prognostic tool of surgical risk in lung resections. The Annals of thoracic surgery. 2002;73(5):1576–81.

67. Pao JJ, Biggs M, Duncan D, Lin DI, Davis R, Huang RS, et al. Predicting EGFR mutational status from pathology images using a real-world dataset. Scientific reports. 2023;13(1):4404.

68. Jiao Y, Xue B, Lu C, Avidan MS, Kannampallil T. Continuous real-time prediction of surgical case duration using a modular artificial neural network. British journal of anaesthesia. 2022;128(5):829–37.

69. Ershad M, Rege R, Majewicz Fey A. Automatic and near real-time stylistic behavior assessment in robotic surgery. International journal of computer assisted radiology and surgery. 2019;14:635–43.

70. Huang AY, Joerger G, Fikfak V, Salmon R, Dunkin BJ, Bass BL, et al. The SmartOR: a distributed sensor network to improve operating room efficiency. Surgical endoscopy. 2017;31:3590–5.

71. Bendixen M, Jørgensen OD, Kronborg C, Andersen C, Licht PB. Postoperative pain and quality of life after lobectomy via video-assisted thoracoscopic surgery or anterolateral thoracotomy for early stage lung cancer: a randomised controlled trial. The Lancet Oncology. 2016;17(6):836–44.

72. Connor CW. Artificial intelligence and machine learning in anesthesiology. Anesthesiology. 2019;131(6):1346.

73. Xu C, Zhu Y, Wu L, Yu H, Liu J, Zhou F, et al. Evaluating the effect of an artificial intelligence system on the anesthesia quality control during gastrointestinal endoscopy with sedation: a randomized controlled trial. BMC anesthesiology. 2022;22(1):313.

74. de Vos J, Visser LA, de Beer AA, Fornasa M, Thoral PJ, Elbers PW, et al. The potential cost-effectiveness of a machine learning tool that can prevent untimely intensive care unit discharge. Value in Health. 2022;25(3):359–67.

75. Lee HK, Jin R, Feng Y, Bain PA, Goffinet J, Baker C, et al. An analytical framework for TJR readmission prediction and cost-effective intervention. IEEE journal of biomedical and health informatics. 2018;23(4):1760–72.

76. Eigner I, Bodendorf F, editors. Decision support for patient discharge in hospitals–analyzing the relationship between length of stay and readmission risk, cost, and profit. Services–SERVICES 2020: 16th World Congress, Held as Part of the Services Conference Federation, SCF 2020, Honolulu, HI, USA, September 18-20, 2020, Proceedings 16; 2020: Springer.

77. Ericson O, Hjelmgren J, Sjövall F, Söderberg J, Persson I. The potential cost and cost-effectiveness impact of using a machine learning algorithm for early detection of sepsis in intensive care units in Sweden. Journal of health economics and outcomes research. 2022;9(1):101.

78. Calvert J, Hoffman J, Barton C, Shimabukuro D, Ries M, Chettipally U, et al. Cost and mortality impact of an algorithm-driven sepsis prediction system. Journal of medical economics. 2017;20(6):646–51.

79. Zwerwer LR, van der Pol S, Zacharowski K, Postma MJ, Kloka J, Friedrichson B, et al. The value of artificial intelligence for the treatment of mechanically ventilated intensive care unit patients: An early health technology assessment. Journal of critical care. 2024;82:154802.

80. Aanesen M, Moilanen M, Olsen F. Economic gains from electronic message exchange: The importance of working procedures. International journal of medical informatics. 2010;79(9):658–67.

81. Mourad M, Cucina R, Ramanathan R, Vidyarthi AR. Addressing the business of discharge: building a case for an electronic discharge summary. Journal of hospital medicine. 2011;6(1):37–42.

82. Sales B, Machado L, Moraes R. Interactive collaboration for virtual reality systems related to medical education and training. Technology and Medical Sciences. 2011;2011:157–62.

83. Tsai M-D, Liu C-S, Liu H-Y, Hsieh M-S, Tsai F-C, editors. Virtual reality facial contouring surgery simulator based on CT transversal slices. 2011 5th International Conference on Bioinformatics and Biomedical Engineering; 2011: IEEE.

84. Cecil J, Pirela-Cruz M, editors. An information model for designing virtual environments for orthopedic surgery. OTM Confederated International Conferences" On the Move to Meaningful Internet Systems"; 2013: Springer.

85. Paranjape K, Schinkel M, Panday RN, Car J, Nanayakkara P. Introducing artificial intelligence training in medical education. JMIR medical education. 2019;5(2):e16048.

86. Torner J, Skouras S, Molinuevo JL, Gispert JD, Alpiste F. Multipurpose virtual reality environment for biomedical and health applications. IEEE Transactions on Neural Systems and Rehabilitation Engineering. 2019;27(8):1511–20.

87. Gupta A, Cecil J, Pirela-Cruz M, Ramanathan P. A virtual reality enhanced cyber-human framework for orthopedic surgical training. IEEE Systems Journal. 2019;13(3):3501–12.

88. Wiljer D, Hakim Z. Developing an artificial intelligence–enabled health care practice: rewiring health care professions for better care. Journal of medical imaging and radiation sciences. 2019;50(4):S8–S14.

89. Deist TM, Dankers FJ, Ojha P, Marshall MS, Janssen T, Faivre-Finn C, et al. Distributed learning on 20 000+ lung cancer patients–The Personal Health Train. Radiotherapy and Oncology. 2020;144:189–200.

90. Jumelle AKL, Ispas I, Thuernmler C, Mival OH, Kosta E, Casla P, et al., editors. Ethical assessment in e-Health. 2014 IEEE 16th International Conference on e-Health Networking, Applications and Services (Healthcom); 2014: IEEE.

91. Antoniou ZC, Panayides AS, Pantzaris M, Constantinides AG, Pattichis CS, Pattichis MS. Real-time adaptation to time-varying constraints for medical video communications. IEEE journal of biomedical and health informatics. 2017;22(4):1177–88.

92. Jaiman V, Urovi V. A consent model for blockchain-based health data sharing platforms. IEEE access. 2020;8:143734–45.
