# Supplementary material for: Unveiling the Benefits of Artificial Intelligence in Individual, Organizational Management, and the Health/Sector System
Source: Health Sci Rep. 2025 Dec 27;9(1):e71536. doi: 10.1002/hsr2.71536 (PMC12743724; doi:10.1002/hsr2.71536)
Supplement: Supplementary file 2 — Supplementary Material 1. [file HSR2-9-e71536-s001.docx]

Supplementary material table Search strings used in Databases

| Database | Search String |
| --- | --- |
| PubMed | ("artificial intelligence"[MeSH Terms] OR "artificial intelligence"[Title/Abstract] OR "machine learning"[Title/Abstract]) AND ("individual"[Title/Abstract] OR "personal"[Title/Abstract] OR "organizational"[Title/Abstract] OR "management"[Title/Abstract] OR "health sector"[Title/Abstract] OR "healthcare"[Title/Abstract]) |
| Scopus | \| TITLE-ABS-KEY("artificial intelligence" OR "machine learning") AND TITLE-ABS-KEY("individual" OR "personal" OR "organizational" OR "management" OR "health sector" OR "healthcare") |
| Web of Science | TS=("artificial intelligence" OR "machine learning") AND TS=("individual" OR "personal" OR "organizational" OR "management" OR "health sector" OR "healthcare") |
| Emerald Insight | ("artificial intelligence" OR "machine learning") AND ("individual" OR "personal" OR "organizational" OR "management" OR "health sector" OR "healthcare") |
